# Risk rates and profiles at intake in child and adolescent mental health services: A cohort and latent class analyses of 21,688 young people in South London

**DOI:** 10.1002/jcv2.12246

**Published:** 2024-05-17

**Authors:** Barry Coughlan, Matt Woolgar, Rick Hood, Dustin Hutchinson, Ella Denford, Amy Hillier, Keith Clements, Teresa Geraghty, Ava Berry, Paul Bywaters, Andy Bilson, Jack Smith, Taliah Drayak, David Graham, Francesca Crozier‐Roche, Robbie Duschinsky

**Affiliations:** ^1^ Department of Public Health and Primary Care University of Cambridge Cambridge UK; ^2^ Institute of Psychiatry, Psychology and Neuroscience King's College London London UK; ^3^ Department of Social Work and Social Care Kingston University London UK; ^4^ National Children's Bureau London UK; ^5^ School of Sport, Exercise and Health Sciences Loughborough Univeristy Loughborough UK; ^6^ Department of Psychology Univerity of Bath Bath UK; ^7^ School of Human and Health Sciences University of Huddersfield Huddersfield UK; ^8^ School of Social Work, Care and Community University of Central Lancashire Preston UK; ^9^ The Care Leavers Association Manchester UK

**Keywords:** adversity, CAMHS, mental health, risk factors, safeguarding

## Abstract

**Background:**

Children and young people (CYP) seen by child and adolescent mental health services (CAMHS) often experience safeguarding issues. Yet little is known about the volume and nature of these risks, including how different adversities or risks relate to one another. This exploratory study aims to bridge this gap, examining rates at entry to services and profiles of risk using a latent class analysis.

**Methods:**

Data were extracted for CYP who received at least one risk assessment at CAMHs in South London between January 2007 and December 2017. In total, there were 21,688 risk assessments. Latent class analysis was used to identify profiles of risk from the risk assessments.

**Results:**

Concerns about parent mental health (*n* = 5274; 24%), emotional abuse (*n* = 4487; 21%), violence towards others (*n* = 4210; 19%), destructive behaviour (*n* = 4005; 18%), and not attending school (*n* = 3762; 17%) were the most commonly identified risks. Six distinct profiles of risk were identified from the latent class analyses: (1) maltreatment and externalising behaviours, (2) maltreatment but low risk to self and others, (3) antisocial behaviour, (4) inadequate caregiver supervision and risk to self and others, (5) risk to self but not others, and (6) mental health needs but low risk.

**Conclusions:**

These findings provide fresh insights into adverse experiences and risks identified by CAMHS. For professionals, the profiles identified in this study might provide insights into profiles of identified risks, in contrast to traditional cumulative approaches to risk. For researchers, these profiles may be fertile ground for hypothesis‐driven work on the association between adversity and later outcomes.

**Key points**

- Despite constituting an important clinical activity, few empirical studies have examined representations of risk in child and adolescent mental health services (CAMHS).
- The most frequently identified risk in CAMHS were: parental mental health (24%), emotional abuse (21%), violence towards others (19%), destructive behaviour (%), and not attending school (17%).
- We identified six profiles of risk (1) maltreatment and externalising behaviours, (2) maltreatment but low risk to self and others, (3) antisocial behaviour, (4) inadequate caregiver supervision and risk to self and others, (5) risk to self but not others, and (6) mental health needs but low risk.
- Risk conceptualisations may benefit from the inclusion of measures of economic disadvantage.

## INTRODUCTION

Many children and young people (CYP) seen by child and adolescent mental health services (CAMHS) experience safeguarding needs, based on circumstances that are directly harmful or that contribute to risky behaviour by CYP towards themselves or others. CAMHS professionals are required to provide an account of these risks using a combination of formal or informal assessment (Aggett & Messent, 2019). Much of the research on risk in CAMHS has focused on developing and sometimes evaluating structured assessment tools (Tiffin et al., 2015). However, the profile of the risks identified by routine CAMHS practice has remained understudied.

This is somewhat surprising because there is strong meta‐analytic evidence to show that risks experienced by CYP are associated with various adverse outcomes, especially mental ill‐health (Hailes et al., 2019; Sahle et al., 2021). Yet there remains considerable uncertainty regarding the most useful ways of conceptualising and modelling these risks. Perhaps the dominant approach in research and practice is to assess cumulative adversity or risk, with various safety threats summed as a quantitative total; this is the strategy adopted, for instance, by the widely used Adverse Childhood Experiences (ACEs) measure (Felitti et al., 1998).

However, disadvantages of the cumulative approach include that it treats a diversity of risks as equivalent to one another, and does not explore their interrelations. One emergent but increasingly common approach, which addresses these concerns, is latent class modelling. In contrast to traditional variable centred approaches, latent class models aim to identify distinct sub‐groups of CYP based on patterns of responses in observed or manifest variables.

These approaches are increasingly used to model risk in developmental science (e.g., Lian et al., 2022) and in children's social care (e.g., Anthony et al., 2023; Hood et al., 2023). These studies have identified a complex range of need profiles that transcend conventional cumulative models, providing a more thorough explanation of risk in these populations.

This study aims to contribute to our understanding of risk practices in CAMHS in two key ways. First, it aims to provide a descriptive account of the different risks identified in CAMHS risk assessments. Second, using latent class analysis, this study aims to identify and distinguish different typologies of risk identified by CAMHS. Like other work using this latent class approach (e.g., Anthony et al., 2023; Hood et al., 2023) this work is exploratory and aims to generate hypotheses for future studies. With this in mind, the current study is oriented by two exploratory research questions:

(1) What is the prevalence of different risks identified in CAMHS?
(2) What are the risk profiles assessed by CAMHS?

## METHOD

### Study population

Data were extracted for 21,688 CYP attending CAMHS in South London and the Maudsley NHS Foundation Trust (SLaM) who had a risk assessment between January 2007 and December 2017. SLaM is one of the largest mental health providers in Europe, with a catchment area of over 1.3 million people and covers four London boroughs: Croydon, Lambeth, Southwark and Lewisham (Perera et al., 2016). Data were extracted using the Clinical Record Interactive Search (CRIS). Further details including a cohort profile of CRIS are provided by Perera et al. (2016).

### Measures

The risk assessment that is the focus of this paper is called the ‘brief risk assessment’ and was used routinely by CAMHS in SLaM between 2007 and 2017. The brief risk assessment is a bespoke assessment which was developed by clinicians based on practice‐based evidence. We are not aware of previous attempts to establish the psychometric properties of the scale. Assessment of risk is a complex process, one influenced by many factors, and it can be anticipated that the CAMHS risk assessment may differ in some ways from the risk assessment that would be conducted by children's social care, or the perception of risk of the young person themselves.

There was also a longer risk assessment that was used during this period which will be the subject of another study. In 2014 a new risk assessment superseded the brief risk assessment; however the brief risk assessment remained in use until 2017.

On examination of the items of the risk assessment, there is perhaps some distinction between a group of items that signal perceived exposure of CYP to maltreatment, and a group of risks where the CYP is regarded as a agent of risk to themselves or others. However, this distinction is not sharp, with some items that do not fit well; it is not clear whether this distinction was intended by the authors of the assessment. It can be anticipated that all the items form part of a single risk assessment because they constitute safeguarding concerns with a bearing on the CYP's mental health and treatment. Furthermore, consultations with experts‐by‐experience and recent research (e.g., Firmin et al., 2022) has highlighted that in practice young people's risks to themselves and others are often not considered as distinct from other safeguarding issues such as threats outside the home. A decision was therefore made to analyse all the items in the risk assessment together, rather than deciding to make an a priori division.

It was not uncommon for CYP to have more than one risk assessment. Here we present analysis of the first risk assessment in each record, in order that our findings might speak to the dilemmas for clinicians and mental health services in understanding and planning for young people's safeguarding needs at intake. This resulted in *n* = 21,688 initial risk assessments. Sociodemographic characteristics of the cohort are presented in Table 1. Age was estimated by calculating the difference between truncated date of birth and date of first risk assessment. Gender is a structured field in the data; due to small cell sizes and in line with the CRIS security model to preserve patient anonymity we could not present data on CYP who were not identified as male or female. Truncated postcodes were extracted and used to estimate deprivation using the index of multiple deprivation (IMD) approach (Department for Communities and Local Government, 2015). Following standard practice, IMD was grouped into quintiles with the first quintile representing the most deprived and the fifth representing the lowest level of deprivation.

**TABLE 1:** Sociodemographic characteristics.

#	*N*	%	Class 1: Maltreatment and externalising behaviours (*n* = 1025)	%	Class 2: Maltreatment but low risk to self and others (*n* = 2620)	%	Class 3: Antisocial behaviour (*n* = 2758)	%	Class 4: Inadequate caregiver supervision and risk to self and others (*n* = 932)	%	Class 5: Risk to self but not others (*n* = 1799)		Class 6: Mental health needs but low risk (*n* = 12,554)	%
Female	9369	43.20	461	44.98	1365	52.1	635	23.02	300	32.19	1106	61.44	5502	43.81
Male	12319	56.80	564	55.02	1255	47.9	2123	76.95	632	67.81	693	38.5	7052	56.15
White	11572	53.36	500	48.78	1224	46.72	1426	51.72	512	54.94	1029	57.22	6881	54.81
Mixed	1956	9.02	106	10.34	309	11.79	287	10.4	104	11.16	144	8	1006	8.03
Asian	1069	4.93	43	4.2	117	4.47	83	3.01	23	2.47	104	5.78	699	5.57
Black	6185	28.52	335	32.68	841	32.1	882	31.97	267	28.65	443	24.61	3417	27.21
Another	906	4.18	41	4	129	4.92	80	2.9	26	2.79	79	4.39	551	4.39
0–3 years	1034	4.77	21	2.05	195	7.44	54	1.96	‐	‐	11	0.61	753	6
4–11 years	9335	43.04	384	37.46	1226	46.79	1482	53.75	126	13.52	286	15.89	5831	46.46
12–17 years	11319	52.19	620	60.49	1199	45.76	1222	44.29	806	86.48	1502	83.5	5970	47.54
1st Quintile	8030	37.03	426	41.56	1048	40	1160	42.04	353	37.88	644	35.83	4399	35.03
2nd Quintile	7134	32.89	339	33.07	938	35.8	905	32.84	343	36.8	578	32.11	4031	32.1
3rd Quintile	3737	17.23	183	17.85	427	16.3	406	14.72	144	15.45	308	17.11	2269	18.08
4th Quintile	1711	7.89	48	4.68	136	5.19	190	6.89	65	6.97	156	8.67	1116	8.89
5th Quintile	1076	4.96	29	2.83	71	2.71	97	3.52	27	2.9	113	6.28	739	5.88
CPP not indicated	16917	78.00	564	55.02	1580	60.31	2133	77.35	582	62.45	1420	78.94	10638	84.74
CPP past or present	4771	22.00	461	44.98	1040	39.69	625	22.65	350	37.55	379	21.06	1916	15.26

*Note*: Cell sizes too small to present CYP not identified as male or female separately.

Abbreviation: CPP, Child protection plan.

### Overview of statistical analysis

Descriptive statistics were estimated for the full sample. A series of latent class analyses (LCAs) were estimated and goodness of fit (AIC/BIC) statistics used to determine the most appropriate number of classes. We then estimated the sociodemographic profiles for each of the classes. Data were cleaned and analysed in R version 4.2.2 (R Core Team, 2022). A full list of R packages used for the analysis is presented in Supplement (S1).

Several steps were required to prepare the data for latent class analyses. The first task was to ensure all variables were at the same level of measurement. In the risk assessment, variables such as carer substance misuse and carer mental ill‐health are binary (i.e., yes or no) and others, for example, self‐harm and risk‐taking, are ordinal (i.e. no risk, low risk, medium risk, high risk, not known). In their authoritative text on LCA, Nylund‐Gibson and Choi (2018) highlight that LCA is generally conducted with binary data and indeed provide an example in which ordinal data are dichotomised in preparation for the LCA. Following Nylund Gibson and Choi's example, we dichotomised ordinal variables into binary variables. Thus, all variables in the LCA were at the same level of measurement. Following discussions with clinical colleagues and following LCA coding conventions (i.e. 1 as the starting value), we aggerated the labels “no risk”, “low risk”, and “not known” were coded as 1 (i.e. low risk/not known) and “medium risk” and “high risk” (medium/high risk) were coded as 2.

Correlation matrices were estimated for each of the risk assessment to investigate redundancy (i.e. items measuring the same construct). We did not identify any item pairings above *r* = 0.70. The only association above *r* = 0.60 was between violence and destructive behaviour (*r* = 0.63), which we regarded as related but distinct types of antisocial behaviour (Burt, 2012). The full correlation matrix for all items is presented in the Supplement (S2).

Following best practice conventions (see Nylund‐Gibson & Choi, 2018) we adopted a stepwise approach to increasing the number of classes by an interval of one. This stepwise approach enabled us to monitor changes in model fit and identify when improvements to model fit plateaued. The point of overparameterization and diminishing decrement acted as the threshold for estimating classes. Model fit was assessed by examination of relative entropy and the following information criterion: Akaike Information Criterion (AIC), Bozdogan's criterion (CAIC), Bayesian Information Criterion (BIC). Based on the item response probability, profile plots were created for each class.

As a response is required by the service for all risk items, no missing data were identified in the risk fields or for gender. The only variables with missing data were ethnicity (6.7%) and deprivation (1.1%). Missing data were imputed using multiple imputation by chained equation (MICE). Given the data are categorical we used a random forest approach and 10 multiple imputations.

Subgroup analysis was conducted on gender, age, ethnicity, IMD and child protection status following the method outlined in the glca vignette (Kim et al., 2022). We examined whether each covariate had an impact on the class prevalence in a model with constrained item response probabilities. Then we ran the model again without constraining item‐response probabilities. Constrained and unconstrained models were then compared using chi‐square analysis. This allowed us to examine whether there were statistically significant (*p* < 0.001) differences in the measurement model (e.g. was the measurement model different for males vs. females). Logistic regression was then used to explore the association between class membership and sociodemographic and child protection characteristics.

### Ethical approval

Ethical approval for CRIS to be used for secondary data analysis is provided by the National Research Ethics Committee South Central Oxford C (ref: 23/SC/0257), subject to approvals from the CRIS Oversight committee for individual projects. The CRIS Oversight Committee is comprised of professionals and experts‐by‐experience. The current project was approved by the CRIS research oversight committee in March 2021 (21–028).

### Experts‐by‐Experience

Facilitated by the National Children's Bureau (NCB), three groups of experts‐by‐experience (EbyE) were consulted. We sought expert‐by‐experience interpretation of the items of the risk assessment, and feedback on an initial interpretation of the study findings. These groups of experts by experience were (1) care experienced CYP, (2) disabled young people with experience of social care involvement, and (3) parents of CYP who have had social care involvement.

## RESULTS

### Sociodemographic characteristics and risk rates

21,688 CYP had at least one risk assessment in CAMHS between January 2007 and December 2017. This cohort had a mean age of 11.1 years (standard deviation = 4.22) and a median age of 12 years. Further details of the sociodemographic characteristics and risk profiles are presented in Table 1. Details of risk rates reported in Table 2.

**TABLE 2:** Frequency of identified risk items.

Risk items	Full cohort (*n* = 21688)		Female (*n* = 9369)		Male (*n* = 12319)		White (*n* = 11572)		Mixed (*n* = 1956)		Asian (*n* = 1069)		Black (*n* = 6185)		Other (*n* = 906)		0–11 years (*n* = 10369)		12–17 years (*n* = 11319)	
	N	%	N	%	N	%	N	%	N	%	N	%	N	%	N	%	N	%	N	%
Parent mental health	5274	24	2493	27	2781	23	3079	27	573	29	188	18	1219	20	215	24	2606	25	2668	24
Parent substance misuse	2552	12	1211	13	1341	11	1568	14	338	17	60	6	510	8	76	8	1239	12	1313	12
Emotional abuse (victim)	4487	21	2269	24	2218	18	2233	19	469	24	192	18	1402	23	191	21	1910	18	2577	23
Physical abuse (victim)	2639	12	1199	13	1440	12	1206	10	261	13	130	12	914	15	128	14	1062	10	1577	14
Sexual abuse (victim)	1192	5	849	9	343	3	658	6	103	5	44	4	339	5	48	5	352	3	840	7
Neglect or lack supervision	2806	13	1302	14	1504	12	1308	11	305	16	110	10	977	16	106	12	1281	12	1525	13
Domestic violence	2694	12	1189	13	1505	12	1329	11	345	18	107	10	804	13	109	12	1423	14	1271	11
Gang crime (victim)	912	4	295	3	617	5	369	3	94	5	43	4	365	6	41	5	93	1	819	7
Culture of violence	1628	8	579	6	1049	9	711	6	174	9	90	8	591	10	62	7	498	5	1130	10
Violence towards others	4210	19	1127	12	3083	25	2234	19	423	22	141	13	1269	21	143	16	1995	19	2215	20
Destructive behaviour	4005	18	1110	12	2895	24	2162	19	426	22	117	11	1179	19	121	13	1901	18	2104	19
Offending	1377	6	382	4	995	8	696	6	133	7	45	4	458	7	45	5	205	2	1172	10
Dangerous behaviour	2295	11	672	7	1623	13	1232	11	247	13	68	6	670	11	78	9	1027	10	1268	11
Physical abuse (perpetrator)	1480	7	426	5	1054	9	778	7	154	8	47	4	452	7	49	5	611	6	869	8
Exclusion from school	3581	17	1038	11	2543	21	1720	15	370	19	107	10	1282	21	102	11	1227	12	2354	21
Sexual abuse (perpetrator)	412	2	122	1	290	2	204	2	33	2	11	1	153	2	11	1	134	1	278	2
Neglect to self	2598	12	1629	17	969	8	1614	14	184	9	179	17	518	8	103	11	526	5	2072	18
Substance misuse	1270	6	641	7	629	5	757	7	125	6	45	4	290	5	53	6	28	0	1242	11
Not attending school	3762	17	1880	20	1882	15	2309	20	353	18	172	16	805	13	123	14	669	6	3093	27
Running away	1641	8	757	8	884	7	856	7	168	9	65	6	477	8	75	8	527	5	1114	10
Self‐harm	3344	15	2170	23	1174	10	2003	17	285	15	203	19	708	11	145	16	616	6	2728	24
Risk taking behaviour	3637	17	1562	17	2075	17	2004	17	347	18	138	13	1024	17	124	14	1237	12	2400	21

### Latent class analysis

Eight LCAs were estimated for the full sample. Model fit and specification, including relative entropy, is described in Table 3. Each model was compared on maximum log‐likelihood, information criteria AIC, CAIC, BIC, and relative entropy. As per Table 3, no global minimum was identified (i.e. the information criterion continuously reduced with the addition of each class). However, improvements in model fit tended to plateau around the six and seven model. Following visual inspection of the model and discussion with the research team, the main difference between the six class model and the seven class model was that the seven class model split the antisocial behaviour class. Another difference was that relative entropy was slightly higher for the six‐class model than the seven class model (see Table 3). Therefore, the six‐class model was selected for interpretation. Profile plots for the seven class model can be found in the Supplement (S3). Cross tabulations for the 6 class according to sociodemographic and child protection characteristics are presented in Table 1.

**TABLE 3:** Model fit and relative entropy.

*k*	Loglik	Gsq	df	AIC	CAIC	BIC	Entropy
2	−148008.93	62019.94	21649	296107.85	296512.17	296467.17	0.86
3	−143436.03	52874.15	21626	287008.06	287619.03	287551.03	0.86
4	−140354.2	46710.48	21603	280890.39	281708.01	281617.01	0.85
5	−138909.56	43821.22	21580	278047.13	279071.4	278957.4	0.84
**6**	**−138081.08**	**42164.26**	**21557**	**276436.17**	**277667.09**	**277530.09**	**0.84**
7	−137499.62	41001.33	21534	275319.24	276756.82	276596.82	0.83
8	−136966.48	39935.06	21511	274298.97	275943.19	275760.19	0.82

Abbreviations: AIC, Akaike information criterion; BIC, Bayesian information criterion; CAIC, Bozdogan's criterion; df, residual degrees of freedom; Gsq, likelihood‐ratio/deviance statistic; *k*, Number of classes; loglik, maximum log‐likelihood.

### Interpreting the full sample LCA

**FIGURE 1:**
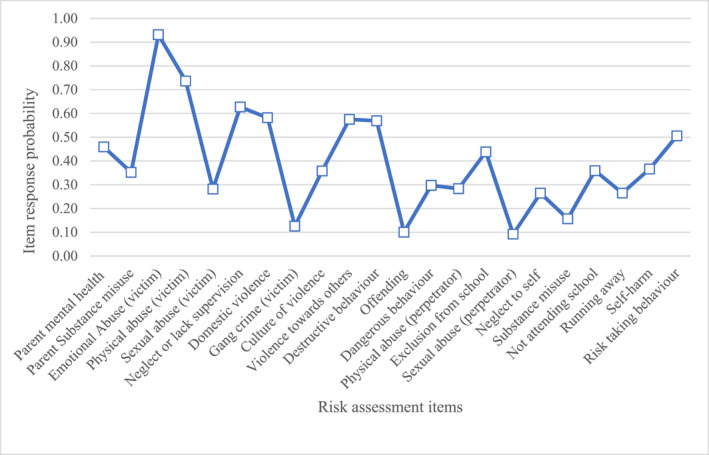
Maltreatment and externalising behaviours (*n* = 1025).

**FIGURE 2:**
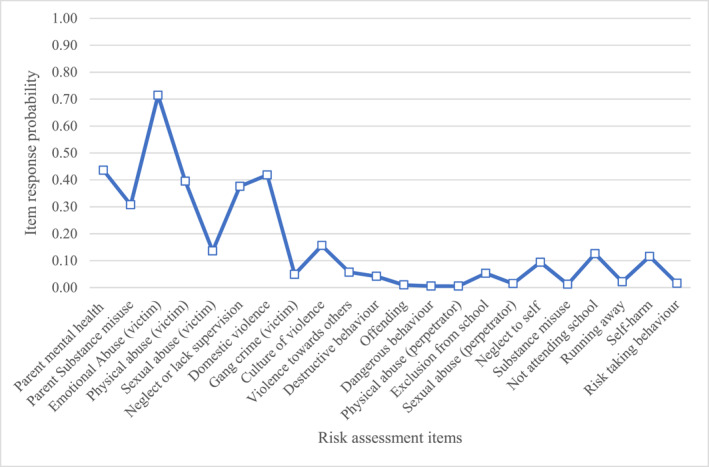
Maltreatment but low risk to self and others (*n* = 2620).

**FIGURE 3:**
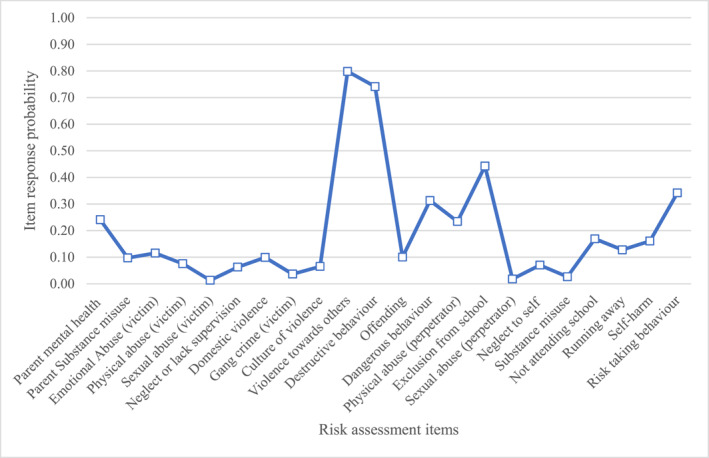
Antisocial behaviour (*n* = 2758).

**FIGURE 4:**
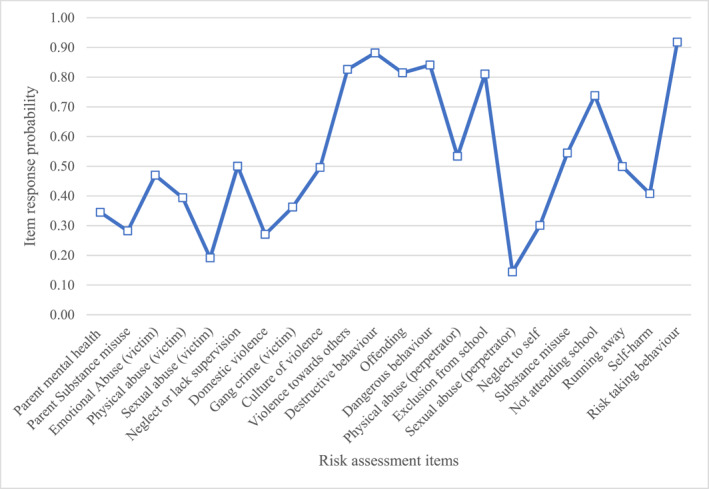
Inadequate caregiver supervision and risk to self and others (*n* = 932).

**FIGURE 5:**
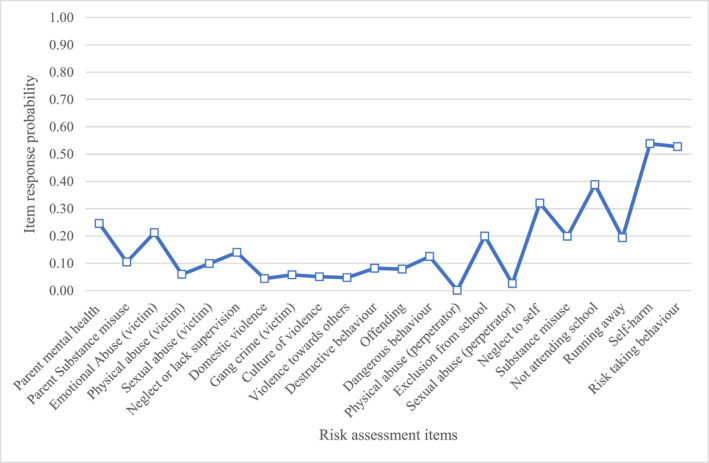
Risk to self but not others (*n* = 1799).

**FIGURE 6:**
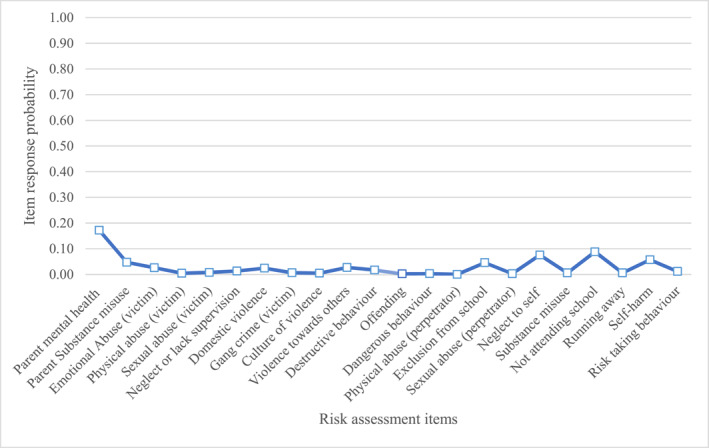
Mental health needs but low risk (*n* = 12,554).

Below are descriptions of the item response probabilities for items in each class. We have provided graphs (Figures 1, 2, 3, 4, 5, 6) and a narrative description of these item‐response probabilities in each case. Item‐response probabilities refer to the likelihood of a particular factor being recorded in a given Class and are presented in brackets in the narrative summary.

#### Class one: Maltreatment and externalising behaviours (*n* = 1025)

Item responses probabilities for Class one are presented in Figure 1. Overall, CYP in Class one had an increased likelihood of being perceived of as at risk for various forms of maltreatment being recorded on the assessments: emotional abuse (0.93), physical abuse (0.74), sexual abuse (0.28), neglect or lack of supervision (0.63), and domestic violence (0.58). Compared to other groups there is a heightened propensity in this group of multiple co‐occurring risk factors, including parental mental health and substance misuse. Although relatively prevalent across most of the classes, parental mental health difficulties (0.46) and parental substance misuse (0.35) were identified marginally more often in this group. Regarding extrafamilial adversities, CYP in this group were identified as at increased risk of gang (0.13) and culture of violence(0.36) relative to CYP perceived as low risk. Clinicians also observed that these CYP were at risk of engaging in externalising and anti‐social behaviours, as indicated by relatively high scores for violence towards others (0.58), destructive behaviour (0.57), dangerous behaviour (0.30), risk‐taking (0.51), and perpetrating physical abuse (0.28). However, it is noteworthy that this group were perceived to not be much at risk of offending (0.10), of misusing substances (0.16) or of perpetrating sexual violence (0.09). Clinicians also described these CYP as being at risk of neglect to themselves (0.26), running away (0.27), and self‐harm (0.37). It is notable that these three risks tended to co‐occur in our data; it might be that they can be conceptualised as forms of internalising behaviour with safeguarding implications. CYP in this group were more likely to have been excluded from school (0.44) or otherwise not be attending school (0.36).

Overall, clinicians considered CYP in this group as at high risk for experiencing various forms of maltreatment. Difficulties in school were a core characteristic of this group. On a behavioural level, clinicians considered these CYP as at heightened risk of harming themselves and others but were not deemed to be at a particularly high risk of offending.

#### Class two: Maltreatment but low risk to self and others (*n* = 2620)

Item responses probabilities for Class two are presented in Figure 2. CYP in Class two had heightened propensity for being identified as at risk for emotional abuse (0.71), physical abuse (0.40) and domestic violence (0.42) and the third highest risk group for sexual abuse (0.14) and neglect (0.38). Like Class one, clinicians described parental mental health difficulties and substance misuse as comparatively common features of these CYP's risk profiles with probabilities of 0.44 and 0.31 respectively. Although there were occasional concerns regarding culture of violence(0.16), there were relatively few concerns regarding gang violence (0.05).

These CYP were not regarded as at particular risk of engaging in behaviours that might harm themselves or others, with item response probabilities ranging between 0.01 and 0.13 for the remaining items. One potential interpretation of this finding is that these CYP are perceived as resilient by clinicians: despite experiences of child maltreatment, and mental health needs sufficient to meet the high threshold for CAMHS involvement, they do not display externalising or internalising behaviours that have safeguarding implications.

#### Class three: Antisocial behaviour (*n* = 2758)

Item response probabilities for CYP in Class three are presented in Figure 3. CYP in Class three had relatively low item‐response probabilities for emotional abuse (0.12), physical abuse (0.08), sexual abuse (0.1), neglect (0.06), and domestic violence (0.10). Similarly, clinicians did not consider parental mental health difficulties (0.24), parental substance misuse (0.10), gang (0.04) or culture of violence(0.07) as particularly strong areas of concern. Instead, the most noteworthy feature of this profile is the high response probabilities for antisocial and externalising behaviours. That is, clinicians perceived CYP in this group to be at heightened risk of violence towards others (0.80), destructive behaviour (0.74), dangerous behaviour (0.31), risk‐taking (0.34), and perpetrating physical abuse (0.23). Like Class one, CYP in this group were characterised as high risk for school exclusion (0.44) and not attending school (0.17). Another similarity is that CYP in this group were not deemed to be high risk for offending (0.10), substance misuse (0.03) or perpetrating sexual violence (0.02). However, unlike Class one, CYP in Class three were not at high risk of neglect to themselves (0.07), running away (0.13), or self‐harm (0.16).

In short, the risk profile of Class three is characterised by concerns regarding antisocial and externalising behaviour. However, unlike for Class one these concerns are not in the context of concerns about maltreatment or extrafamilial threats.

#### Class four: Inadequate caregiver supervision and risk to self and others (*n* = 932)

Item response probabilities for Class four are presented in Figure 4. CYP in Class four were identified as the second highest group for neglect or lack of supervision (0.50) and sexual abuse (0.19). They were also the third highest risk group for emotional abuse (0.47), physical abuse (0.39) and domestic violence (0.27). This group was considered highest risk for exposure to gang (0.36) and culture of violence (0.50). Additionally, like Class one and Class two, parental mental health (0.34) and substance misuse (0.28) were relatively frequently reported by clinicians for CYP in this group. Of all groups, CYP in Class four were considered at highest risk for externalising and antisocial behaviours with increased item‐response probabilities of violence towards others (0.83), destructive behaviour (0.88), dangerous behaviour (0.84), risk‐taking (0.92), and perpetrating physical (0.53) and sexual (0.14) abuse of others. Perhaps as a result, even more so than other groups, clinicians had concerns regarding school exclusion (0.81) and otherwise not attending school (0.74). Further, concerns regarding offending (0.81), running away (0.50), substance misuse (0.54), self‐neglect (0.30), self‐harm (0.41) were more prevalent in this group than any other group.

Overall these CYP appear to be regarded as at heightened risk of family dysfunction, especially neglect and lack of supervision. This in turn may increase exposure to extra‐familial harm or cultural violence. More than any other group, clinicians report that these CYP have difficulties engaging with school and are at high risk of harming themselves and others and becoming involved in the criminal justice system.

#### Class five: Risk to self but not others (*n* = 1799)

Item response probabilities for CYP in Class five are presented in Figure 5. Class five were identified as relatively low risk for all examined forms of maltreatment, with item‐response probabilities ranging between 0.04 (domestic violence) and 0.21 (emotional abuse). Similarly, clinicians had relatively few concerns regarding parental mental health difficulties (0.25), parental substance misuse (0.10), and gang (0.06) or culture of violence (0.05). Clinicians reported very few concerns about externalising or anti‐social behaviours such as violence (0.05) or destructive behaviour (0.08). However, of all groups, Class five were deemed to be at the highest risk for self‐harm (0.54). They were also often regarded as showing risk‐taking behaviours (0.53), neglect to self (0.32), substance misuse (0.20). And running away (0.19). Perhaps related to these risk‐taking behaviours, school exclusion was relatively common (0.20) and they were the second highest risk group in terms of perceived likelihood of not attending school (0.39).

Overall CYP in Class five were of concern to clinicians for issues around self‐harm, self‐neglect, and difficulties attending school, rather than due to external sources of threat to the CYP or the likelihood of externalising behaviour.

#### Class six: Mental health needs but low risk (*n* = 12,554)

Item response probabilities for CYP in Class six are presented in Figure 6. Class six was by far the largest group, representing somewhat over half of all cases seen by CAMHS. In contrast to the other groups of patients, CYP in Class six are characterised as relatively low risk on all items. Overall, the highest item‐response probability in this class was identified for parental mental health difficulties (0.17).

It seems that Class six represents the large group of CYP who have mental health needs sufficient to have been referred to CAMHS, but who are perceived as low risk by clinicians.

### Subgroup analysis

Chi‐square analysis of deviance suggested that the measurement invariance assumption was violated for sociodemographic and child protection characteristics. Therefore, separate LCAs were estimated for each subgroup. Overall, a six class model was indicated for most, but not all subgroups. Specifically, a six class model was not the optimum fit for: Asian ethnicity (five class model); other ethnicity (four class model); 0–3 years of age (three class model); IMD 4^th^ quintile (five class model); and IMD 5th quintile (five class model). Details of model fit and profile plots for all subgroups can be found in the Supplement (S4–S36).

Multinomial logistic regression was used to estimate the associations between sociodemographic characteristics, including child protection involvement and class membership. These analyses were conducted on the subset of the data (*n* = 16,299) for whom a 6 class model was identified as the best fit. Therefore, participants who had the following characteristics were excluded from this part of the analysis: Asian ethnicity; other ethnicity; 0–3 years of age; IMD 4th quintile; and IMD 5th quintile. Model fit and profile plots for the subset of the data are presented in Supplement (S37 and S38). For the multinomial logistic regression, Class six (Mental health needs but low risk) was used as the reference point ‐ with acknowledgement that this group are not a representative community sample. For covariates, the generalised variance inflation factor was below 3 and Cramer's V were below 0.25, suggesting that there is weak or limited multicollinearity among the covariates.

The results of this analysis are presented in Table 4. Gender differences can be seen in several of the analyses. For instance, compared to the low risk group, classes three (Antisocial Behaviour) and four (Inadequate caregiver supervision and risk to self and others) were more likely to be male. In contrast, CYP in classes two (Maltreatment but low risk to self and others) and five (Risk to self but not others) were more likely to be female. Regarding ethnicity, CYP in classes one (Maltreatment and externalising behaviours), two (Maltreatment but low risk to self and others), three (Antisocial behaviour) and four (Inadequate caregiver supervision and risk to self and others) were more likely to be identified as mixed ethnicity. CYP assigned to class two (Maltreatment but low risk to self and others) were more likely to be identified as black. Significant associations between age and class membership were identified, with CYP in classes one (Maltreatment and externalising behaviours), for (Inadequate caregiver supervision and risk to self and others) and five (Risk to self but not others) being more frequent in the older age range (12–17). In comparison to the low risk group (Class six), the risk of assignment in classes one through five reduced as deprivation decreased. Classes one through five were also more likely to have child protection involvement, compared to the low risk group (Class six).

**TABLE 4:** Multinomial logistic regression, showing associations between sociodemographic/child protection characteristics and latent class membership.

Subgroup	Class one: Maltreatment and externalising behaviours (*n* = 848)				Class two: Maltreatment but low risk to self and others (*n* = 2019)				Class three: Antisocial behaviour (*n* = 2270)				Class four: Inadequate caregiver supervision and risk to self and others (*n* = 795)				Class five: Risk to self but not others (*n* = 1350)			
	RR	CI (L)	CI (U)	*p*	RR	CI (L)	CI (U)	*p*	RR	CI (L)	CI (U)	*p*	RRR	CI (L)	CI (U)	*p*	RRR	CI (L)	CI (U)	*p*
Male	1.05	0.91	1.22	0.50	**0.66**	**0.60**	**0.74**	**<0.00**	**2.64**	**2.37**	**2.95**	**<0.00**	**2.58**	**2.19**	**3.04**	**<0.00**	**0.64**	**0.57**	**0.72**	**<0.00**
Ethnicity: mixed	**1.29**	**1.01**	**1.64**	**0.04**	**1.46**	**1.24**	**1.71**	**<0.00**	**1.24**	**1.06**	**1.45**	**0.01**	**1.37**	**1.08**	**1.75**	**0.01**	1.05	0.85	1.29	0.64
Ethnicity: black	1.12	0.95	1.31	0.17	**1.16**	**1.04**	**1.29**	**0.01**	1.09	0.99	1.21	0.08	0.97	0.82	1.14	0.71	0.96	0.84	1.10	0.55
12–17 years	**1.90**	**1.63**	**2.21**	**<0.00**	1.02	0.92	1.13	0.69	1.05	0.96	1.16	0.30	**7.95**	**6.48**	**9.76**	**<0.00**	**4.68**	**4.02**	**5.44**	**<0.00**
IMD 2	**0.82**	**0.70**	**0.97**	**0.02**	0.96	0.87	1.08	0.52	**0.86**	**0.77**	**0.95**	**<0.00**	0.98	0.83	1.16	0.83	0.96	0.84	1.09	0.50
IMD 3	**0.75**	**0.62**	**0.92**	**<0.00**	**0.76**	**0.66**	**0.87**	**<0.00**	**0.71**	**0.62**	**0.81**	**<0.00**	**0.70**	**0.57**	**0.87**	**<0.00**	**0.85**	**0.72**	**1.00**	**0.04**
CPP	**4.61**	**3.97**	**5.35**	**<0.00**	**3.41**	**3.06**	**3.79**	**<0.00**	**1.67**	**3.08**	**4.25**	**<0.00**	**3.62**	**3.08**	**4.25**	**<0.00**	**1.69**	**1.46**	**1.96**	**<0.00**

*Note*: Bold = statistically significant findings.

Abbreviations: CI (L), Confidence interval lower; CI (U), Confidence Interval Upper; *p*, *p* value; RR, relative risk ratio.

## DISCUSSION

This exploratory study provides some intriguing insights about the rates and typologies of risk recorded by CAMHS. One notable finding was that the rates of parental mental health difficulties found in this study (24%) are comparable, not only to the rates documented in children's social care assessments (25.5%; Department for Education, 2022), but also in the rates of parental mental health observed in non‐clinical populations (e.g., 23.3%; Maybery et al., 2009). In fact, these rates are comparable with the prevalence of mental health symptoms among UK adults more generally (17% McManus et al., 2016). Therefore, although it might be expected that a population attending CAMHS would have a higher prevalence of parental mental health, in fact these rates are comparable to other populations.

Given that much has been made of the importance of parental mental health in child safeguarding (e.g., Brandon, 2009), the apparent consistency of parental mental health difficulties across CAMHS, children's social care and non‐clinical populations, calls into question the status of parental mental health as a threat to child safety if not co‐occurring with other difficulties. Indeed, similar to other studies (e.g., Hood et al., 2023), parental mental health in our data was not a sole defining feature in any of the risk profiles, and was even elevated in the low risk group (Class six). One challenge is that parents may not disclose experiences of mental health with clinicians, particularly in the early sessions. Still, these findings also align with Roscoe et al. (2021) recent study of 4070 child welfare decisions, which found that much of the association between parental mental health and child removals could be explained by other safety threats (e.g., failure to meet immediate needs of the child). Therefore, a better understanding of the status of parental mental health in relation to child safety is required to support effective assessment practices. In the meantime, the findings from this study and others (e.g., Roscoe et al., 2021) suggest that parental mental health in and of itself may not be an imminent threat to child safety either in terms of contextual (e.g., maltreatment) or more individual harms (e.g., self‐harm). Instead, in line with previous work (e.g., Hood et al., 2023) it seems that parental mental health seems to increase risk when combined with other factors (e.g., substance misuse).

Another thought‐provoking finding was that half the profiles (classes one, three, four) were characterised by considerable risks around externalising or antisocial behaviours (e.g., violence towards others, destructive behaviour). Yet there were also notable differences between these classes. One difference was the variations in maltreatment. This distinction is sharp between classes one (Maltreatment and externalising behaviours) and three (Antisocial behaviour) and is in line with the findings of Hood et al. (2023) work on social care assessments. The distinction between classes one and four (Inadequate caregiver supervision and risk to self and others) with regard to maltreatment is less clear, however, class one appeared to experience more emotional abuse.

Difficulties engaging with school (either by exclusion on non‐attendance) was a consistent feature across classes one, three and four. Both school exclusion and externalising behaviours are well known risk factors for later involvement in the criminal justice system (Sanders et al., 2020). It might therefore be expected that each of these groups were perceived to be at increased risk for offending. In fact, only class four (Inadequate caregiver supervision and risk to self and others) had a considerably higher propensity for offending. Further, compared to the other externalising groups (classes one and two), class four were also at increased risk of substance misuse and risk‐taking behaviour.

These findings are potentially noteworthy because offending, substance misuse and risk‐taking are all associated with executive functioning (Gustavson et al., 2017; Reynolds et al., 2019; Seruca & Silva, 2016). It is plausible therefore that the distinction between classes one and four may be explained, at least in part, by differences in executive functioning in addition to differences in threats of maltreatment. However, these findings may also reflect the social, cultural and institutional conditions in which clinicians make judgments about who is at risk of offending, for instance extrapolating from assumptions about lack of parental supervision. Further work is planned to explore clinical profiles, including neurodevelopmental differences, and criminal justice involvement with this sample.

Regarding severe internalising problems such as self‐harm, meta‐analyses have documented robust associations between maltreatment and self‐harm (Liu et al., 2018). Therefore, it is noteworthy that we did not observe a class characterised by maltreatment and self‐harm specifically. This may reflect a methodological limitation given this is a risk assessment based upon the first assessment and several lines of evidence suggest that CYP generally experience various barriers to discussing self‐harm with professionals (Waller et al., 2023). We also know that maltreatment can erode trust in others (Neil et al., 2022), a point also raised by our experts‐by‐experience. Our expert‐by‐experience consultations suggested that it may be the case that CYP who experienced maltreatment were less likely to report issues with self‐harm to clinicians early in the initial sessions. To better understand this, further work is now being done examining risk profiles from later assessment.

From a clinical perspective, the findings reported here shed new light on how risk is conceptualised in CAMHS. These risk profiles might provide a more clinically meaningful method of conceptualising threats to child safety than conventional cumulative approaches (e.g., Felitti et al., 1998). An important question, however, is whether these profiles are a more robust predictor of relevant outcomes than cumulative approaches. Further work is already underway examining the associations between the risk profiles identified in this study and various outcomes including later child protection involvement, mental health diagnosis, self‐harm and service provision.

It is crucial to note that certain profiles may not be valid for some groups. For instance, we did not find evidence of Class 5 (risk to self but not others) in children 3 years and younger (see S27). A further issue is that the risk assessment does not include any measures of severe socio‐economic deprivation or insecurity, for example, from poverty, homelessness, having no recourse to public funds, having an insecure immigration status. Subgroup analysis shows a social gradient in conceptualisations of risk. Therefore, it is plausible that were such socio‐economic inequalities included in the assessment the profiles would have been materially different. Furthermore, the absence of contextual factors in the risk assessment may lead to an approach where the CYP or family becomes problematised and the contribution of inequality is ignored. Given the socio‐economic gradient observed in the subgroup analysis we regard this as an important topic for further study and aim to explore in future work whether classes of risk or the cumulative approach predicts clinical outcomes (e.g., diagnosis, service activity) over and above economic deprivation.

### Strengths and limitations

A strength of this study is that it draws from a large clinical cohort of CYP. Further by using data from the risk assessment, which are required fields, we were able to minimise the volume of missing data, a common challenge when working with administrative data. Regarding risk profiles, one source of validation was the finding that all risk groups were at increased risk of having child protection involvement. Further work validating the risk profiles, including a comparison with the cumulative model.

Still, several limitations should also be noted. First, the categories derived from administrative data may have limited reliability and validity because it is not possible to know whether clinicians are using these data fields in the same way and for the same reasons each time. As acknowledged in the Methods, various assessor (e.g., professional background and experience), informant, (e.g., relationship to young person), and service‐related (e.g., limited resources) factors likely influence how different clinicians perceive and assess risk. Meta‐data on these additional factors is not available. In depth qualitative work is underway examining how risk is conceptualised across professional groups. A second limitation, the rates and models here are based on the first risk assessment for each young person. As above, it is plausible that some risks might only come to light later in the CYPs episode of care (e.g., self‐harm in the context of maltreatment). Third, we also do not know what different sources of evidence (e.g., CYP disclosure, parents, social workers etc) clinicians used in their assessment. Third, although improvement in model fit tended to plateau around the six‐class model, no global minimum was identified. Although this is common in LCAs on administrative data (e.g, Anthony et al., 2023; Hood et al., 2023), caution is required when discounting the possibility of a seven class model, details of which can be found in the supplement (S3). Fourth, the risk profiles are dependent on the items that are assessed. It might be the case that were other possible safety threats assessed (e.g., racial discrimination, bullying in school) different profiles might emerge. Fifth, as others have described (e.g., Perera et al., 2016), compared to national averages, SLaM has a considerably higher representation of patients from black minority groups and lower proportion of patients from Asian minority groups. Therefore it would be beneficial to test these models in other parts of the UK. Sixth, structured assessments are just one source of information about how risk is represented. Qualitative research addressing representations of risk in CAMHS clinical notes might be a helpful further study.

## CONCLUSION

This study provides us with fresh insights about the rates and profiles of risk identified by CAMHS. These findings suggest that, at least at entry to services, parental mental health is the most frequently cited concern. Yet there is evidence that concerns about parental mental health on their own are not necessarily indicative of high risk of child maltreatment unless combined with other risks/concerns, such as substance misuse. LCA identified a broad range of externalising presentations which had some notable differences in terms of the role of maltreatment and offending. Distinctions between these profiles and the absence of some profiles (e.g., maltreatment and self‐harm) raise intriguing questions for further studies.

## AUTHOR CONTRIBUTIONS

**Barry Coughlan**: Conceptualization, data curation, formal analysis, funding acquisition, investigation, methodology, project administration, visualization, writing – original draft, writing – review & editing. **Matt Woolgar**: Conceptualization, data curation, investigation, methodology, project administration, supervision, writing – original draft, writing – review & editing. **Rick Hood**: Conceptualization, methodology, supervision, writing – original draft, writing – review & editing. **Dustin Hutchinson**: Conceptualization, methodology, writing – review & editing. **Ella Denford**: Formal analysis, methodology, writing – review & editing. **Amy Hillier**: Formal analysis, methodology, writing – review & editing. **Keith Clements**: Methodology project administration, writing – review & editing. **Teresa Geraghty**: Methodology project administration, writing – review & editing. **Ava Berry**: Methodology project administration, writing – review & editing. **Paul Bywaters**: Conceptualization, methodology, supervision, writing – review & editing. **Andy Bilson**: Conceptualization, methodology, supervision, writing – review & editing. **Jack Smith**: Methodology, writing – review & editing. **Taliah Drayak**: Methodology, writing – review & editing. **David Graham**: Methodology, writing – review & editing. **Francesca Crozier‐Roche**: Methodology, writing – review & editing. **Robbie Duschinsky**: Conceptualization, funding acquisition, methodology, project administration, supervision, writing – original draft, writing – review & editing.

## CONFLICT OF INTEREST STATEMENT

The authors have declared that they have no competing or potential conflicts of interest.

## ETHICS STATEMENT

Ethical approval for CRIS to be used for secondary data analysis is provided by the National Research Ethics Committee South Central Oxford C (ref: 23/SC/0257), subject to approvals from the CRIS Oversight committee for individual projects. The CRIS Oversight Committee is comprised of professionals and experts‐by‐experience. The current project was approved by the CRIS research oversight committee in March 2021 (21–028).

## Supporting information

Supporting Information S1

## Data Availability

Data are owned by a third party, Maudsley Biomedical Research Centre (BRC) Clinical Records. Interactive Search (CRIS) tool, which provides access to anonymised data derived from SLaM electronic medical records. These data can only be accessed by permitted individuals from within a secure firewall (i.e. the data cannot be sent elsewhere), in the same manner as the authors. For more information please contact: cris.administrator@slam.nhs.uk.
