# Supplementary material for: Risk rates and profiles at intake in child and adolescent mental health services: A cohort and latent class analyses of 21,688 young people in South London
Source: JCPP Adv. 2024 May 17;4(3):e12246. doi: 10.1002/jcv2.12246 (PMC11472811; doi:10.1002/jcv2.12246)

Supplementary materials

S1: R packages used

Arnold J (2021). _ggthemes: Extra Themes, Scales and Geoms for 'ggplot2'_. R package version 4.2.4, https://CRAN.R-project.org/package=ggthemes.

Chongsuvivatwong V (2022). _epiDisplay: Epidemiological Data Display Package_. R package version 3.5.0.2, https://CRAN.R-project.org/package=epiDisplay.

Firke S (2023). _janitor: Simple Tools for Examining and Cleaning Dirty Data_. R package version 2.2.0, https://CRAN.R-project.org/package=janitor.

Wickham. ggplot2: Elegant Graphics for Data Analysis. Springer-Verlag New York, 2016.

Wickham (2007). Reshaping Data with the reshape Package. Journal of Statistical Software, 21(12), 1-20. URL, http://www.jstatsoft.org/v21/i12/.

Harrison E, Drake T, Ots R (2023). _finalfit: Quickly Create Elegant Regression Results Tables and Plots when Modelling_. R package version 1.0.6, https://CRAN.R-project.org/package=finalfit.

Henry L, Wickham H (2023). _rlang: Functions for Base Types and Core R and 'Tidyverse' Features_. R package version 1.1.1, https://CRAN.R-project.org/package=rlang.

Kassambara A (2022). _ggcorrplot: Visualization of a Correlation Matrix using 'ggplot2'_. R package version 0.1.4, https://CRAN.R-project.org/package=ggcorrplot.

Kim, Y., Jeon, S., Chang, C., & Chung, H. (2022). glca: An R Package for Multiple-Group Latent Class Analysis. Applied Psychological Measurement, 46(5), 439–441.https://doi.org/10.1177/01466216221084197

Meyer D, Zeileis A, Hornik K (2023). _vcd: Visualizing Categorical Data_. R package version 1.4-11, https://CRAN.R-project.org/package=vcd.

Neuwirth E (2022). _RColorBrewer: ColorBrewer Palettes_. R package version 1.1-3, https://CRAN.R-project.org/package=RColorBrewer.

Slowikowski K (2023). _ggrepel: Automatically Position Non-Overlapping Text Labels with 'ggplot2'_. R package version 0.9.3, https://CRAN.R-project.org/package=ggrepel.

Stef van Buuren, Karin Groothuis-Oudshoorn (2011). mice: Multivariate Imputation by Chained Equations in R. Journal of Statistical Software, 45(3), 1-67. DOI 10.18637/jss.v045.i03.

Taiyun Wei and Viliam Simko (2021). R package 'corrplot': Visualization of a Correlation Matrix (Version 0.92). Available from https://github.com/taiyun/corrplot

Tierney N, Cook D (2023). “Expanding Tidy Data Principles to Facilitate Missing Data Exploration, Visualization and Assessment of Imputations.” _Journal of Statistical Software_, *105*(7), 1-31. doi:10.18637/jss.v105.i07 https://doi.org/10.18637/jss.v105.i07.

Venables, W. N. & Ripley, B. D. (2002) Modern Applied Statistics with S. Fourth Edition. Springer, New York. ISBN 0-387-95457-0

Wickham H, Averick M, Bryan J, Chang W, McGowan LD, François R, Grolemund G, Hayes A, Henry L, Hester J, Kuhn M, Pedersen TL, Miller E, Bache SM, Müller K, Ooms J, Robinson D, Seidel DP, Spinu V, Takahashi K, Vaughan D, Wilke C, Woo K, Yutani H (2019). “Welcome to the tidyverse.” _Journal of Open Source Software_, *4*(43), 1686. doi:10.21105/joss.01686, https://doi.org/10.21105/joss.01686.

Wickham H, François R, Henry L, Müller K, Vaughan D (2023). _dplyr: A Grammar of Data Manipulation_. R package version 1.1.2, https://CRAN.R-project.org/package=dplyr.

S2: Correlation Matrix detailing associations between items


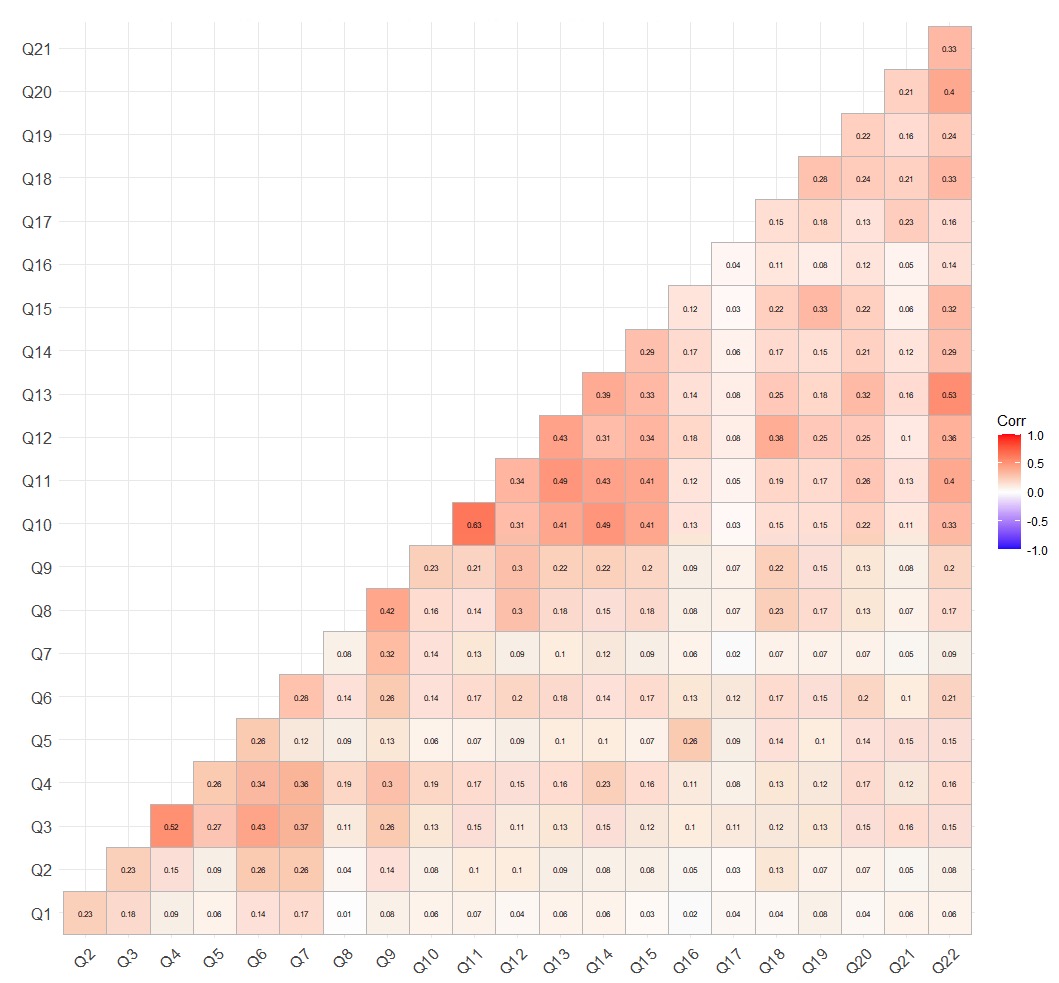

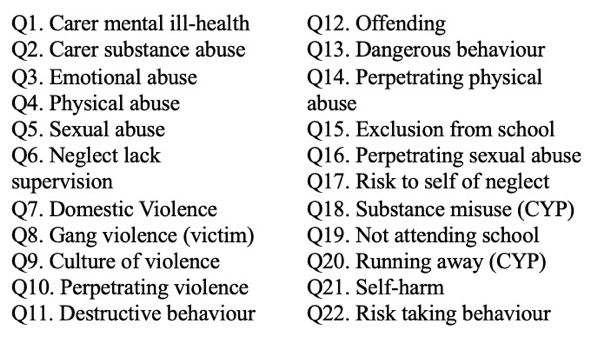


S3. Item-response probabilities for six and seven class models


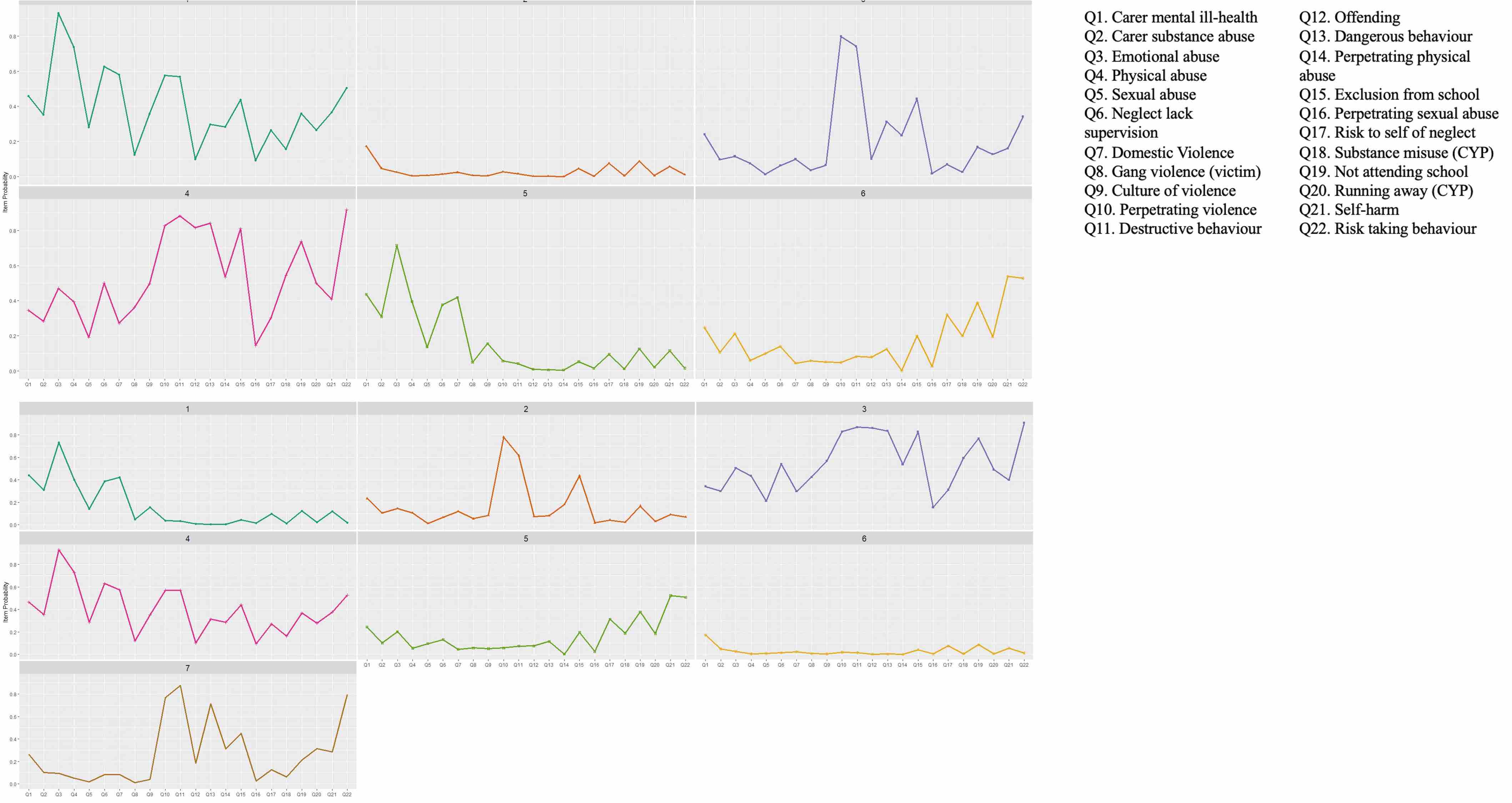


| S4: Model fit female | | | | | | |
| --- | --- | --- | --- | --- | --- | --- |
| k | loglik | Gsq | df | AIC | CAIC | BIC |
| 2 | -63135.91 | 28850.47 | 9323 | 126361.82 | 126728.35 | 126683.35 |
| 3 | -61176.24 | 24931.13 | 9300 | 122488.48 | 123042.35 | 122974.35 |
| 4 | -60042.93 | 22664.52 | 9277 | 120267.87 | 121009.08 | 120918.08 |
| 5 | -59357.81 | 21294.27 | 9254 | 118943.62 | 119872.17 | 119758.17 |
| 6 | -58994.1 | 20566.84 | 9231 | 118262.19 | 119378.08 | 119241.08 |
| 7 | -58808.24 | 20195.12 | 9208 | 117936.47 | 119239.7 | 119079.70 |

k = Number of classes; loglik = maximum log-likelihood; Gsq = likelihood-ratio/deviance statistic; df = residual degrees of freedom; AIC = Akaike information criterion; CAIC = Bozdogan's criterion; BIC = Bayesian information criterion

S5 Model fit Male

| k | loglik | Gsq | df | AIC | CAIC | BIC |
| --- | --- | --- | --- | --- | --- | --- |
| 2 | -82469.31 | 37439.79 | 12273 | 165028.62 | 165407.47 | 165362.47 |
| 3 | -80099.41 | 32699.99 | 12250 | 160334.82 | 160907.3 | 160839.3 |
| 4 | -78114.74 | 28730.65 | 12227 | 156411.48 | 157177.6 | 157086.6 |
| 5 | -77494.58 | 27490.33 | 12204 | 155217.16 | 156176.92 | 156062.92 |
| 6 | -76916.57 | 26334.31 | 12181 | 154107.14 | 155260.53 | 155123.53 |
| 7 | -76518.11 | 25537.38 | 12158 | 153356.21 | 154703.24 | 154543.24 |

k = Number of classes; loglik = maximum log-likelihood; Gsq = likelihood-ratio/deviance statistic; df = residual degrees of freedom; AIC = Akaike information criterion; CAIC = Bozdogan's criterion; BIC = Bayesian information criterion

S6 Model fit White

|  | loglik | Gsq | df | AIC | CAIC | BIC |
| --- | --- | --- | --- | --- | --- | --- |
| 2 | -77964.71 | 36095.82 | 11531 | 156019.43 | 156395.48 | 156350.48 |
| 3 | -75616.04 | 31398.47 | 11508 | 151368.07 | 151936.33 | 151868.33 |
| 4 | -73898.66 | 27963.72 | 11485 | 147979.32 | 148739.79 | 148648.79 |
| 5 | -73089.35 | 26345.09 | 11462 | 146406.69 | 147359.37 | 147245.37 |
| 6 | -72704.86 | 25576.11 | 11439 | 145683.71 | 146828.59 | 146691.59 |
| 7 | -72387.79 | 24941.98 | 11416 | 145095.59 | 146432.67 | 146272.67 |

k = Number of classes; loglik = maximum log-likelihood; Gsq = likelihood-ratio/deviance statistic; df = residual degrees of freedom; AIC = Akaike information criterion; CAIC = Bozdogan's criterion; BIC = Bayesian information criterion

S7 Model fit Mixed

|  | loglik | Gsq | df | AIC | CAIC | BIC |
| --- | --- | --- | --- | --- | --- | --- |
| 2 | -14457.599 | 9063.193 | 1912 | 29005.198 | 29301.284 | 29256.284 |
| 3 | -13954.66 | 8057.316 | 1889 | 28045.321 | 28492.739 | 28424.739 |
| 4 | -13552.819 | 7253.634 | 1866 | 27287.639 | 27886.39 | 27795.39 |
| 5 | -13400.777 | 6949.549 | 1843 | 27029.554 | 27779.638 | 27665.638 |
| 6 | -13280.763 | 6709.521 | 1820 | 26835.526 | 27736.942 | 27599.942 |
| 7 | -13185.725 | 6519.445 | 1797 | 26691.45 | 27744.199 | 27584.199 |

k = Number of classes; loglik = maximum log-likelihood; Gsq = likelihood-ratio/deviance statistic; df = residual degrees of freedom; AIC = Akaike information criterion; CAIC = Bozdogan's criterion; BIC = Bayesian information criterion

| S8 Model fit Asian | | | | | | |
| --- | --- | --- | --- | --- | --- | --- |
|  | loglik | Gsq | df | AIC | CAIC | BIC |
| 2 | -6323.335 | 3936.449 | 1023 | 12736.67 | 13005.521 | 12960.521 |
| 3 | -6155.732 | 3601.243 | 1000 | 12447.464 | 12853.729 | 12785.729 |
| 4 | -6038.643 | 3367.066 | 977 | 12259.287 | 12802.965 | 12711.965 |
| 5 | -5949.165 | 3188.109 | 954 | 12126.33 | 12807.421 | 12693.421 |
| **6** | **-5910.07** | **3109.918** | **931** | **12094.139** | **12912.643** | **12775.643** |
| 7 | -5862.232 | 3014.242 | 908 | 12044.463 | 13000.38 | 12840.38 |

k = Number of classes; loglik = maximum log-likelihood; Gsq = likelihood-ratio/deviance statistic; df = residual degrees of freedom; AIC = Akaike information criterion; CAIC = Bozdogan's criterion; BIC = Bayesian information criterion

S9 Model fit Black

|  | loglik | Gsq | df | AIC | CAIC | BIC |
| --- | --- | --- | --- | --- | --- | --- |
| 2 | -42488.76 | 21731.02 | 6139 | 85067.53 | 85415.37 | 85370.37 |
| 3 | -41069.6 | 18892.7 | 6116 | 82275.2 | 82800.84 | 82732.84 |
| 4 | -40228.18 | 17209.85 | 6093 | 80638.36 | 81341.77 | 81250.77 |
| 5 | -39855.36 | 16464.2 | 6070 | 79938.71 | 80819.92 | 80705.92 |
| 6 | -39553.09 | 15859.68 | 6047 | 79380.19 | 80439.18 | 80302.18 |
| 7 | -39347.18 | 15447.84 | 6024 | 79014.35 | 80251.13 | 80091.13 |

k = Number of classes; loglik = maximum log-likelihood; Gsq = likelihood-ratio/deviance statistic; df = residual degrees of freedom; AIC = Akaike information criterion; CAIC = Bozdogan's criterion; BIC = Bayesian information criterion

| S10 Model fit Other | | | | | | |
| --- | --- | --- | --- | --- | --- | --- |
|  | loglik | Gsq | df | AIC | CAIC | BIC |
| 2 | -5773.528 | 3865.962 | 860 | 11637.056 | 11898.462 | 11853.462 |
| 3 | -5585.962 | 3490.832 | 837 | 11307.925 | 11702.94 | 11634.94 |
| 4 | -5494.603 | 3308.114 | 814 | 11171.207 | 11699.829 | 11608.829 |
| 5 | -5419.362 | 3157.63 | 791 | 11066.723 | 11728.954 | 11614.954 |
| 6 | -5360.084 | 3039.075 | 768 | 10994.169 | 11790.007 | 11653.007 |
| 7 | -5331.203 | 2981.313 | 745 | 10982.406 | 11911.852 | 11751.852 |

k = Number of classes; loglik = maximum log-likelihood; Gsq = likelihood-ratio/deviance statistic; df = residual degrees of freedom; AIC = Akaike information criterion; CAIC = Bozdogan's criterion; BIC = Bayesian information criterion

| S11 Model fit Preschool | | | | | | |
| --- | --- | --- | --- | --- | --- | --- |
|  | loglik | Gsq | df | AIC | CAIC | BIC |
| 2 | -3983.129 | 1927.77 | 988 | 8056.258 | 8323.611 | 8278.611 |
| 3 | -3734.917 | 1431.347 | 965 | 7605.834 | 8009.835 | 7941.835 |
| 4 | -3665.178 | 1291.868 | 942 | 7512.355 | 8053.003 | 7962.003 |
| 5 | -3622.353 | 1206.219 | 919 | 7472.706 | 8150.002 | 8036.002 |
| 6 | -3598.712 | 1158.936 | 896 | 7471.423 | 8285.366 | 8148.366 |
| 7 | -3579.938 | 1121.39 | 873 | 7479.877 | 8430.467 | 8270.467 |

k = Number of classes; loglik = maximum log-likelihood; Gsq = likelihood-ratio/deviance statistic; df = residual degrees of freedom; AIC = Akaike information criterion; CAIC = Bozdogan's criterion; BIC = Bayesian information criterion

| S12 Model fit Primary School | | | | | | |
| --- | --- | --- | --- | --- | --- | --- |
|  | loglik | Gsq | df | AIC | CAIC | BIC |
| 2 | -52266.62 | 20883.26 | 9294 | 104623.25 | 104989.64 | 104944.64 |
| 3 | -49889.88 | 16129.78 | 9271 | 99915.76 | 100469.42 | 100401.42 |
| 4 | -48868.85 | 14087.71 | 9248 | 97919.7 | 98660.62 | 98569.62 |
| 5 | -48516.92 | 13383.86 | 9225 | 97261.84 | 98190.03 | 98076.03 |
| 6 | -48308.92 | 12967.86 | 9202 | 96891.84 | 98007.3 | 97870.3 |
| 7 | -48107.72 | 12565.46 | 9179 | 96535.44 | 97838.17 | 97678.17 |

k = Number of classes; loglik = maximum log-likelihood; Gsq = likelihood-ratio/deviance statistic; df = residual degrees of freedom; AIC = Akaike information criterion; CAIC = Bozdogan's criterion; BIC = Bayesian information criterion

| S13 Model fit Secondary School | | | | | | |
| --- | --- | --- | --- | --- | --- | --- |
|  | loglik | Gsq | df | AIC | CAIC | BIC |
| 2 | -87348.33 | 41925.9 | 11275 | 174786.65 | 175161.7 | 175116.7 |
| 3 | -85122.96 | 37475.16 | 11252 | 170381.92 | 170948.66 | 170880.66 |
| 4 | -83486.93 | 34203.1 | 11229 | 167155.86 | 167914.29 | 167823.29 |
| 5 | -82737.87 | 32704.99 | 11206 | 165703.75 | 166653.87 | 166539.87 |
| 6 | -82202.45 | 31634.15 | 11183 | 164678.91 | 165820.72 | 165683.72 |
| 7 | -81797.82 | 30824.87 | 11160 | 163915.63 | 165249.14 | 165089.14 |

k = Number of classes; loglik = maximum log-likelihood; Gsq = likelihood-ratio/deviance statistic; df = residual degrees of freedom; AIC = Akaike information criterion; CAIC = Bozdogan's criterion; BIC = Bayesian information criterion

| S14 Model fit IMD Quintile 1 | | | | | | |
| --- | --- | --- | --- | --- | --- | --- |
|  | loglik | Gsq | df | AIC | CAIC | BIC |
| 2 | -56991.95 | 28045.91 | 7986 | 114073.89 | 114433.5 | 114388.5 |
| 3 | -55281.44 | 24624.9 | 7963 | 110698.89 | 111242.29 | 111174.29 |
| 4 | -54094.56 | 22251.14 | 7940 | 108371.13 | 109098.32 | 109007.32 |
| 5 | -53567.97 | 21197.94 | 7917 | 107363.93 | 108274.93 | 108160.93 |
| 6 | -53239.23 | 20540.46 | 7894 | 106752.45 | 107847.24 | 107710.24 |
| 7 | -53012.08 | 20086.17 | 7871 | 106344.16 | 107622.75 | 107462.75 |

k = Number of classes; loglik = maximum log-likelihood; Gsq = likelihood-ratio/deviance statistic; df = residual degrees of freedom; AIC = Akaike information criterion; CAIC = Bozdogan's criterion; BIC = Bayesian information criterion

| S15 Model fit IMD Quintile 2 | | | | | | |
| --- | --- | --- | --- | --- | --- | --- |
|  | loglik | Gsq | df | AIC | CAIC | BIC |
| 2 | -49655.29 | 25247 | 7090 | 99400.57 | 99754.85 | 99709.85 |
| 3 | -48013.76 | 21963.96 | 7067 | 96163.53 | 96698.89 | 96630.89 |
| 4 | -46971.66 | 19879.76 | 7044 | 94125.33 | 94841.76 | 94750.76 |
| 5 | -46477.85 | 18892.13 | 7021 | 93183.7 | 94081.21 | 93967.21 |
| 6 | -46191.16 | 18318.76 | 6998 | 92656.32 | 93734.91 | 93597.91 |
| 7 | -45966.52 | 17869.47 | 6975 | 92253.04 | 93512.7 | 93352.7 |

k = Number of classes; loglik = maximum log-likelihood; Gsq = likelihood-ratio/deviance statistic; df = residual degrees of freedom; AIC = Akaike information criterion; CAIC = Bozdogan's criterion; BIC = Bayesian information criterion

| S16 Model fit IMD Quintile 3 | | | | | | |
| --- | --- | --- | --- | --- | --- | --- |
|  | loglik | Gsq | df | AIC | CAIC | BIC |
| 2 | -24595.44 | 13644.998 | 3693 | 49280.879 | 49606.075 | 49561.075 |
| 3 | -23829.588 | 12113.294 | 3670 | 47795.176 | 48286.583 | 48218.583 |
| 4 | -23270.32 | 10994.758 | 3647 | 46722.639 | 47380.257 | 47289.257 |
| 5 | -23031.17 | 10516.458 | 3624 | 46290.34 | 47114.169 | 47000.169 |
| 6 | -22823.186 | 10100.49 | 3601 | 45920.372 | 46910.412 | 46773.412 |
| 7 | -22729.679 | 9913.477 | 3578 | 45779.358 | 46935.61 | 46775.61 |

k = Number of classes; loglik = maximum log-likelihood; Gsq = likelihood-ratio/deviance statistic; df = residual degrees of freedom; AIC = Akaike information criterion; CAIC = Bozdogan's criterion; BIC = Bayesian information criterion

| S17 Model fit IMD Quintile 4 | | | | | | |
| --- | --- | --- | --- | --- | --- | --- |
|  | loglik | Gsq | df | AIC | CAIC | BIC |
| 2 | -10230.788 | 6047.025 | 1666 | 20551.576 | 20841.62 | 20796.62 |
| 3 | -9921.262 | 5427.973 | 1643 | 19978.524 | 20416.812 | 20348.812 |
| 4 | -9690.045 | 4965.539 | 1620 | 19562.09 | 20148.623 | 20057.623 |
| 5 | -9563.305 | 4712.059 | 1597 | 19354.61 | 20089.388 | 19975.388 |
| 6 | -9497.676 | 4580.8 | 1574 | 19269.351 | 20152.373 | 20015.373 |
| 7 | -9440.897 | 4467.243 | 1551 | 19201.794 | 20233.061 | 20073.061 |

k = Number of classes; loglik = maximum log-likelihood; Gsq = likelihood-ratio/deviance statistic; df = residual degrees of freedom; AIC = Akaike information criterion; CAIC = Bozdogan's criterion; BIC = Bayesian information criterion

| S18 Model fit IMD Quintile 5 | | | | | | |
| --- | --- | --- | --- | --- | --- | --- |
|  | loglik | Gsq | df | AIC | CAIC | BIC |
| 2 | -5954.156 | 3548.624 | 1030 | 11998.313 | 12267.458 | 12222.458 |
| 3 | -5778.736 | 3197.784 | 1007 | 11693.472 | 12100.181 | 12032.181 |
| 4 | -5655.075 | 2950.462 | 984 | 11492.15 | 12036.422 | 11945.422 |
| 5 | -5564.805 | 2769.921 | 961 | 11357.609 | 12039.444 | 11925.444 |
| 6 | -5530.261 | 2700.833 | 938 | 11334.522 | 12153.92 | 12016.92 |
| 7 | -5485.065 | 2610.441 | 915 | 11290.129 | 12247.09 | 12087.09 |

k = Number of classes; loglik = maximum log-likelihood; Gsq = likelihood-ratio/deviance statistic; df = residual degrees of freedom; AIC = Akaike information criterion; CAIC = Bozdogan's criterion; BIC = Bayesian information criterion

| S19 Model fit no child protection involvement | | | | | | |
| --- | --- | --- | --- | --- | --- | --- |
|  | loglik | Gsq | df | AIC | CAIC | BIC |
| 2 | -104655.67 | 41835.61 | 16878 | 209401.34 | 209794.49 | 209749.49 |
| 3 | -102001.04 | 36526.35 | 16855 | 204138.09 | 204732.17 | 204664.17 |
| 4 | -100005.41 | 32535.09 | 16832 | 200192.83 | 200987.85 | 200896.85 |
| 5 | -98896.62 | 30317.5 | 16809 | 198021.23 | 199017.19 | 198903.19 |
| 6 | -98383.02 | 29290.32 | 16786 | 197040.05 | 198236.95 | 198099.95 |
| 7 | -97942.66 | 28409.59 | 16763 | 196205.32 | 197603.16 | 197443.16 |

k = Number of classes; loglik = maximum log-likelihood; Gsq = likelihood-ratio/deviance statistic; df = residual degrees of freedom; AIC = Akaike information criterion; CAIC = Bozdogan's criterion; BIC = Bayesian information criterion

| S20 Model fit past or present child protection involvement | | | | | | |
| --- | --- | --- | --- | --- | --- | --- |
|  | loglik | Gsq | df | AIC | CAIC | BIC |
| 2 | -41509.87 | 24747.47 | 4725 | 83109.73 | 83445.9 | 83400.9 |
| 3 | -40010.07 | 21747.88 | 4702 | 80156.15 | 80664.13 | 80596.13 |
| 4 | -39168.99 | 20065.72 | 4679 | 78519.98 | 79199.78 | 79108.78 |
| 5 | -38759.71 | 19247.15 | 4656 | 77747.41 | 78599.03 | 78485.03 |
| 6 | -38418.15 | 18564.05 | 4633 | 77110.31 | 78133.74 | 77996.74 |
| 7 | -38237.08 | 18201.89 | 4610 | 76794.16 | 77989.41 | 77829.41 |

k = Number of classes; loglik = maximum log-likelihood; Gsq = likelihood-ratio/deviance statistic; df = residual degrees of freedom; AIC = Akaike information criterion; CAIC = Bozdogan's criterion; BIC = Bayesian information criterion

S21 Profile plots female


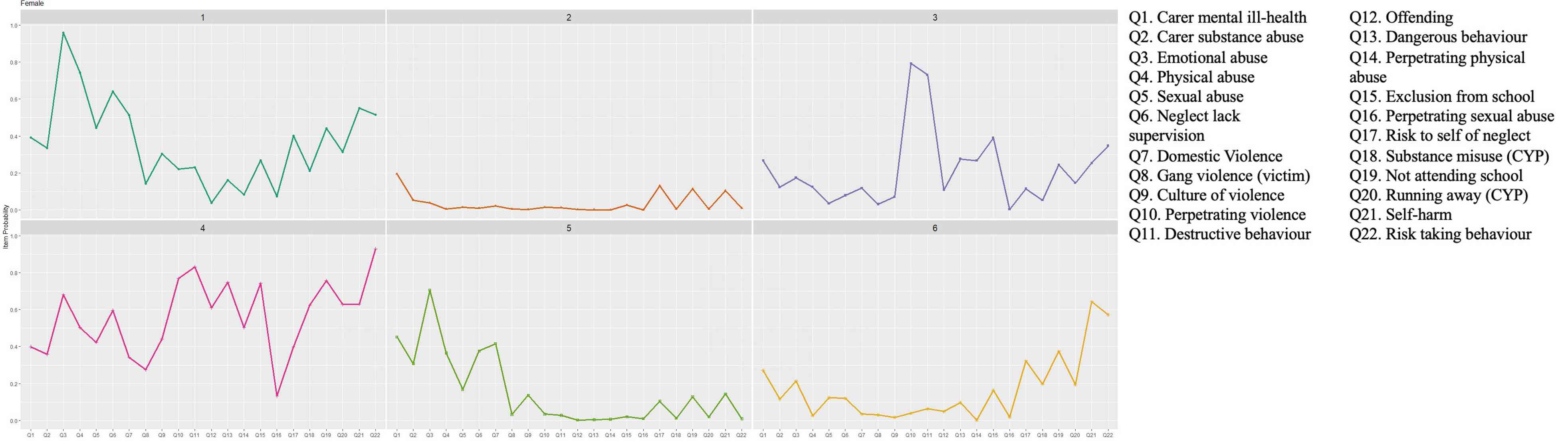


S21 Profile Plots male


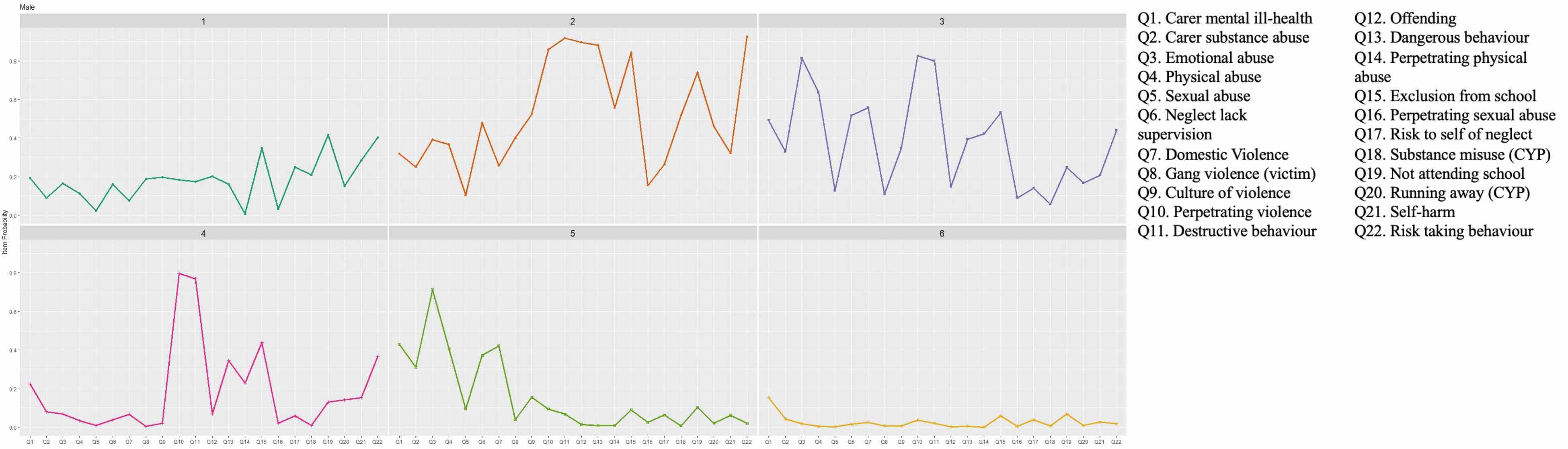


S22 Profile plots white


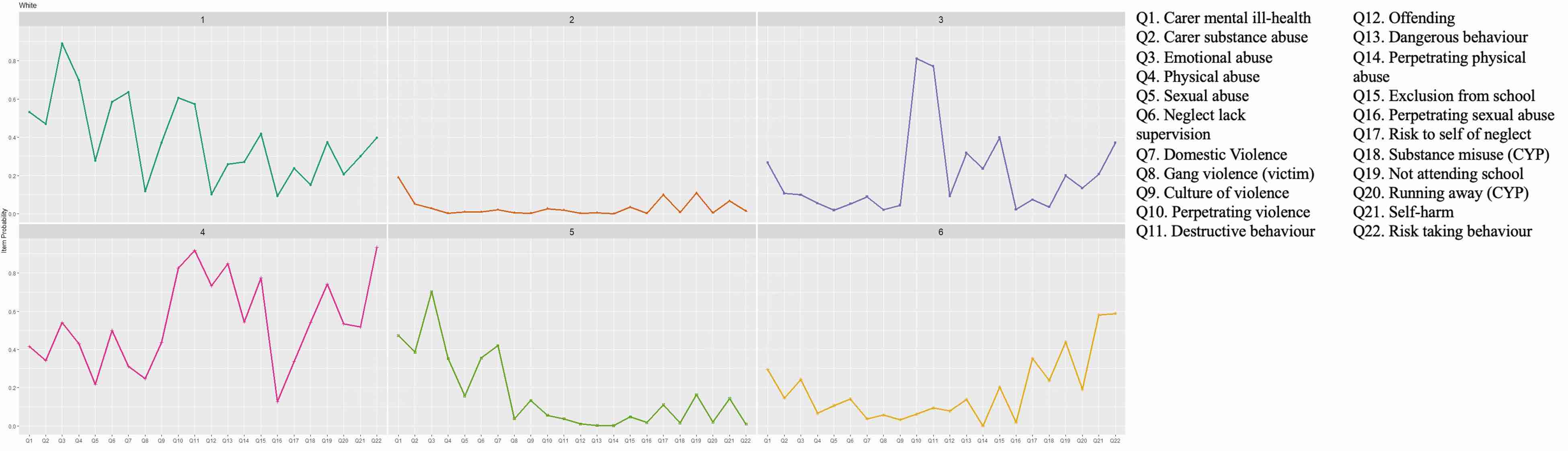


S23 Profile plots mixed race


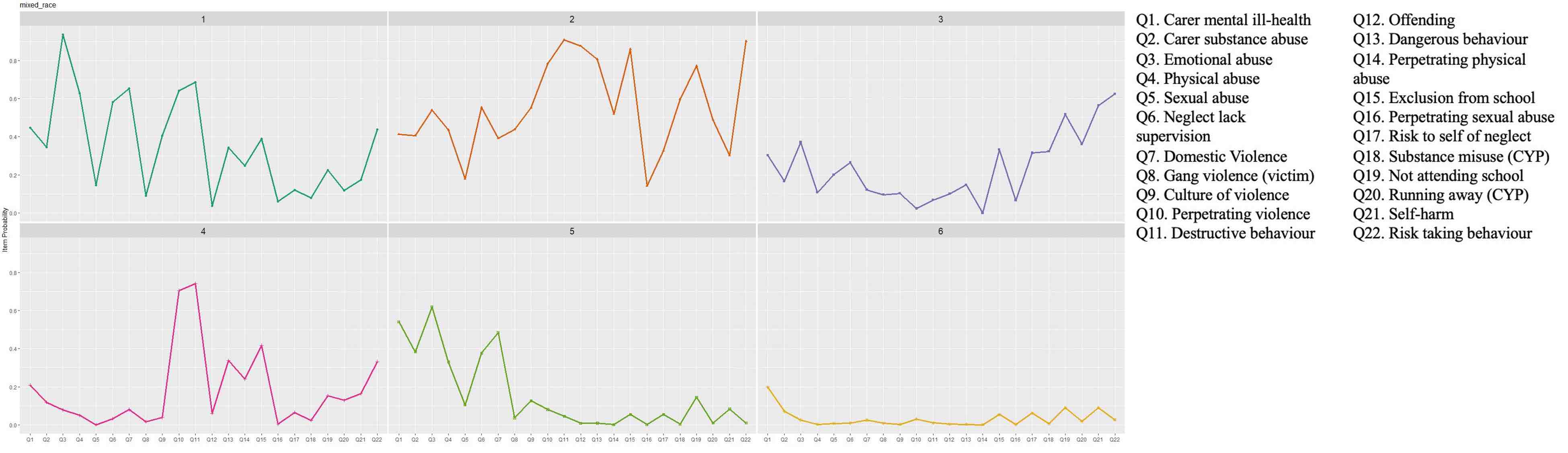


S24 Profile plots Asian


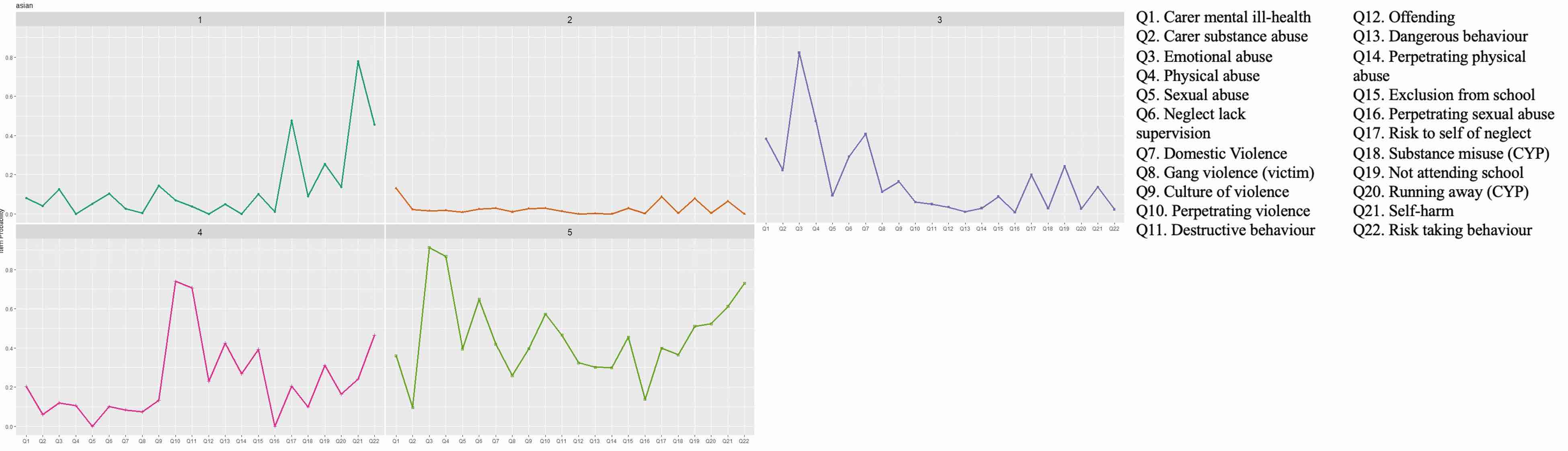


S25 Profile plots black


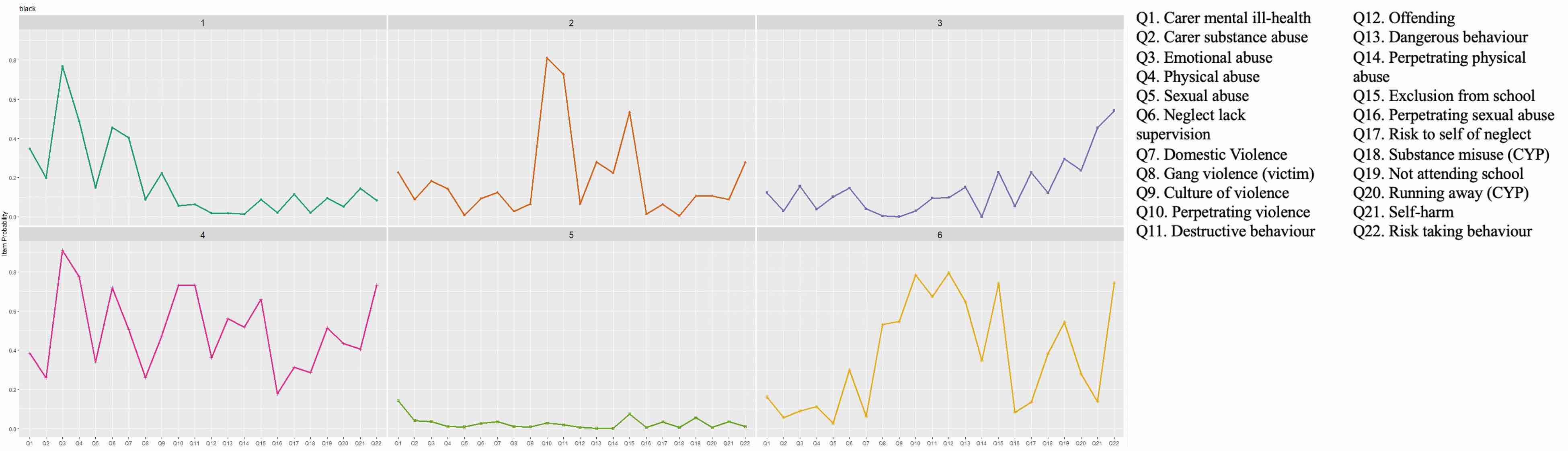


S26 Profile plots Other


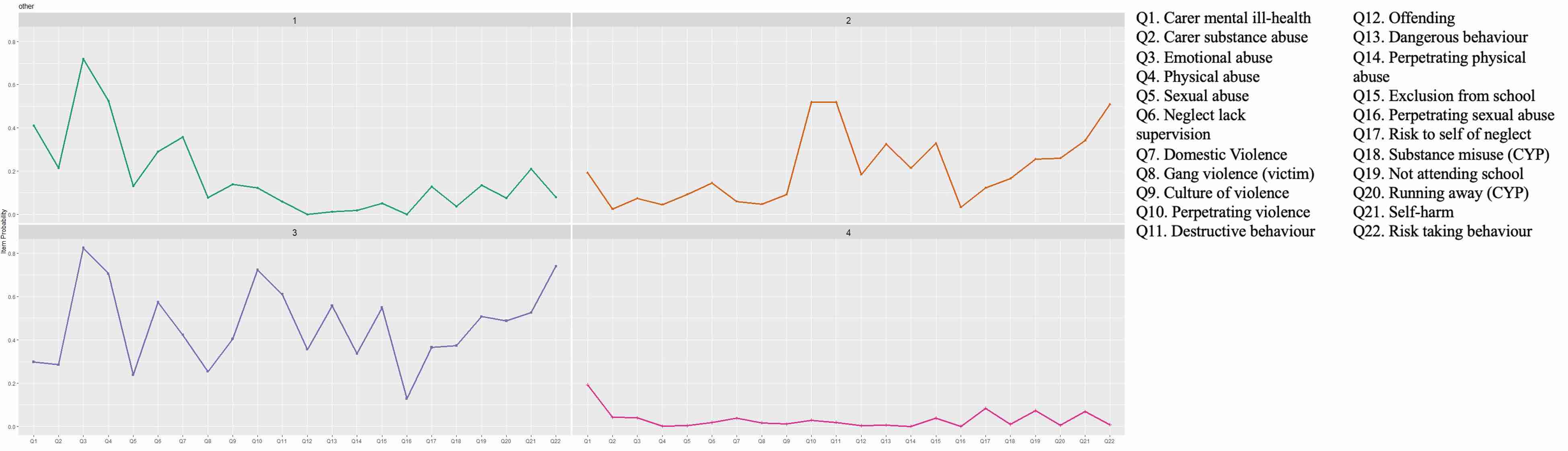


S27 Profile plots Preschool


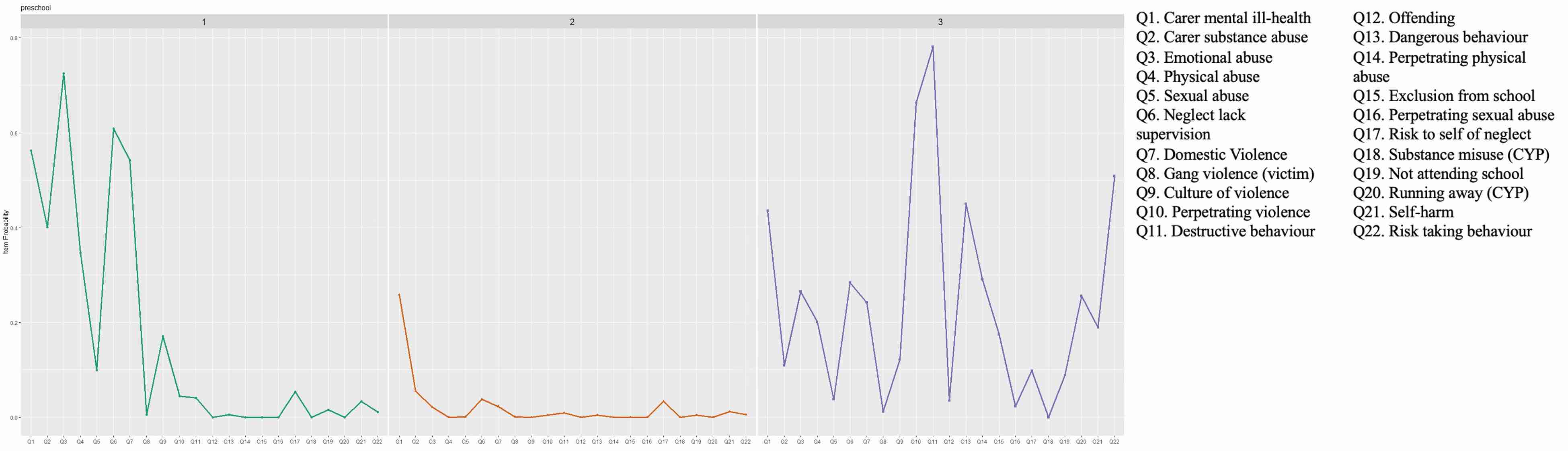


S28 Profile plots Primary School


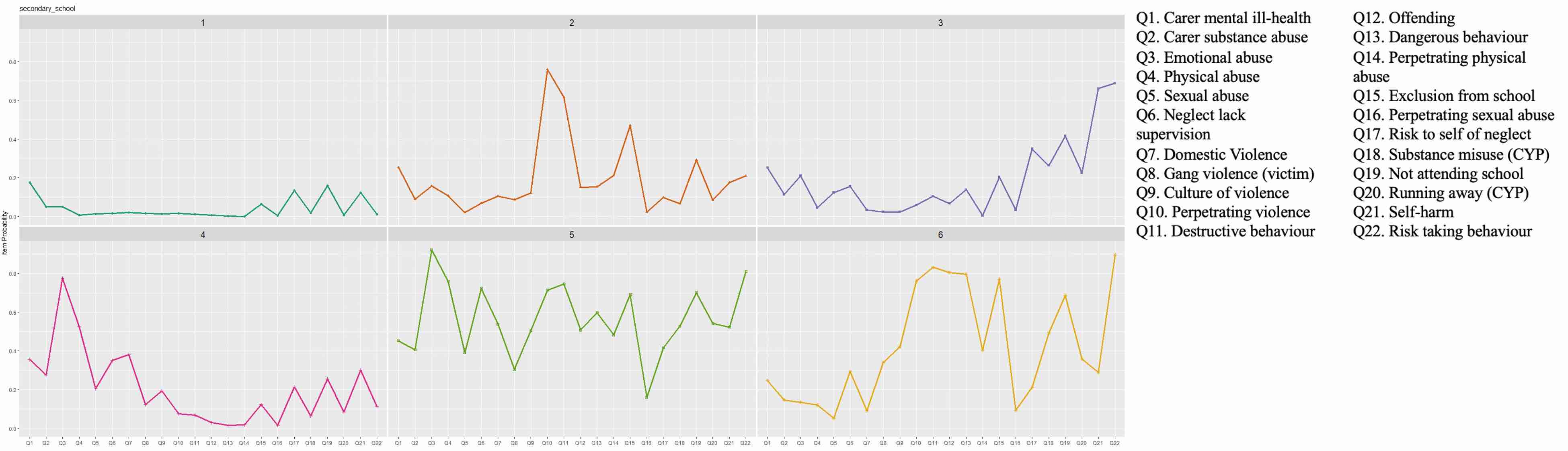


S29 Profile plots Secondary School


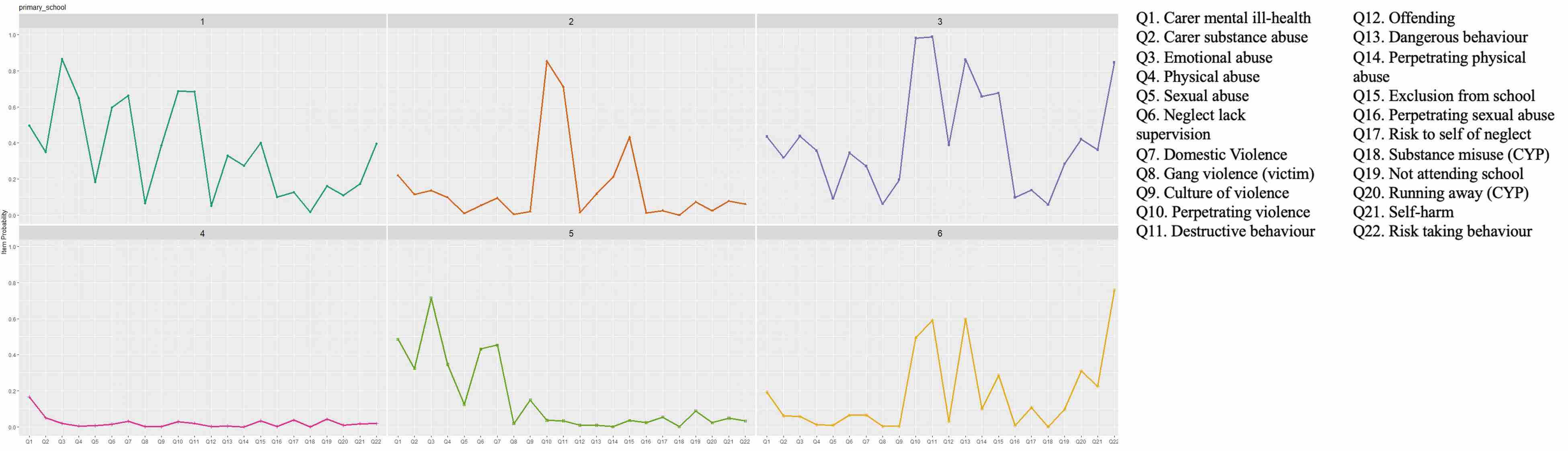


S30 Profile plots IMD Quintile 1^st^


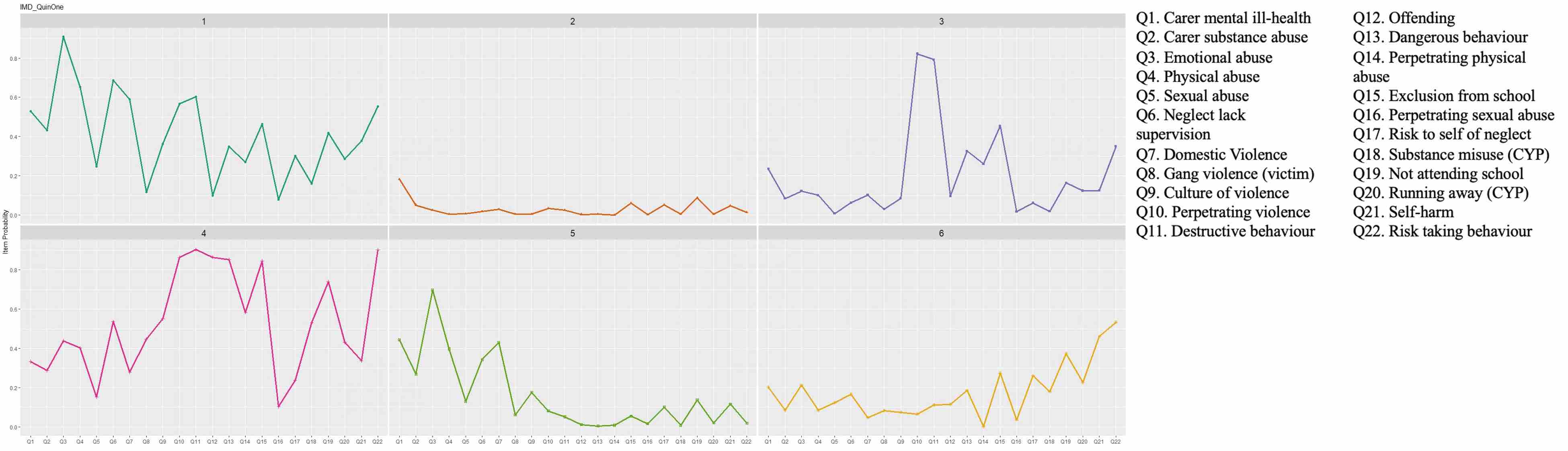


S31 Profile plots IMD Quintile 2^nd^


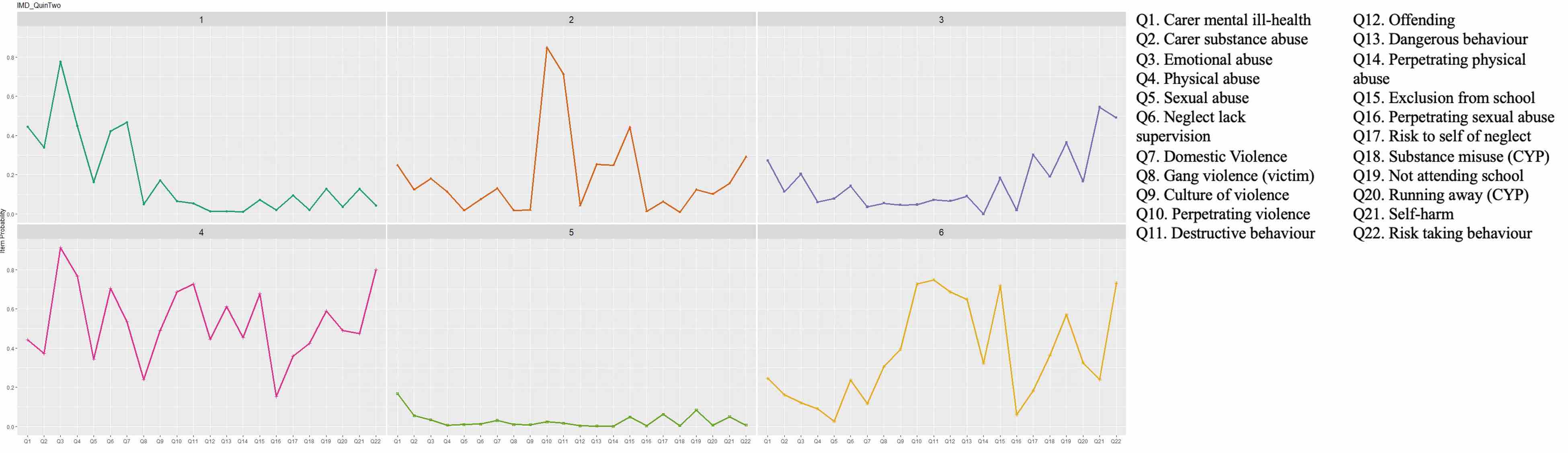


S32 Profile plots IMD Quintile 3^rd^


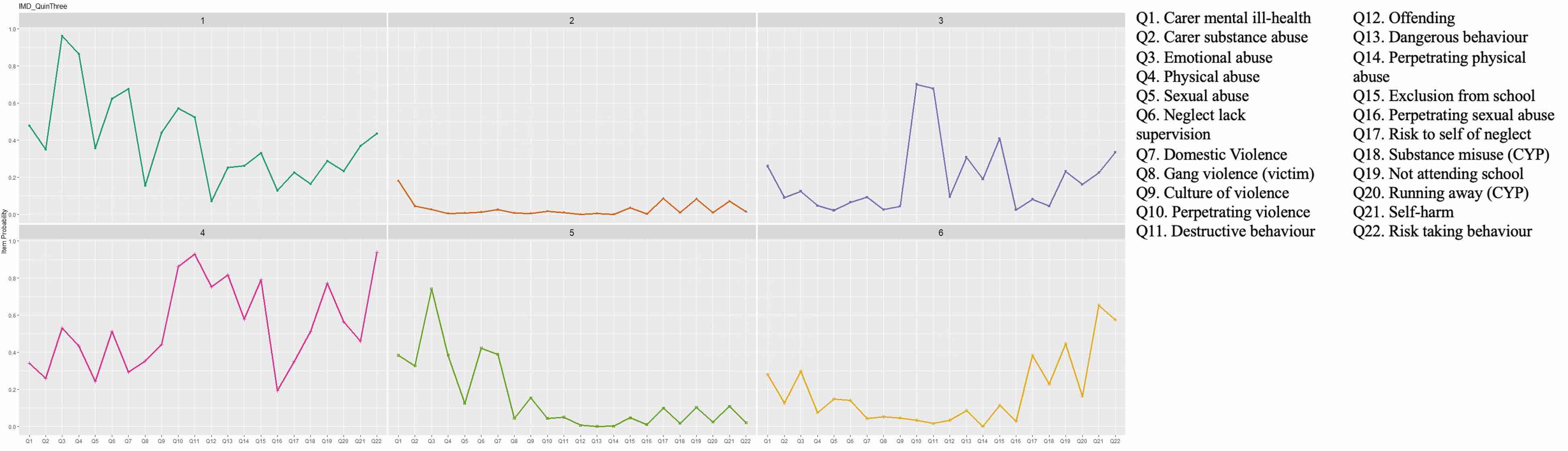


S33 Profile plots IMD Quintile 4^th^


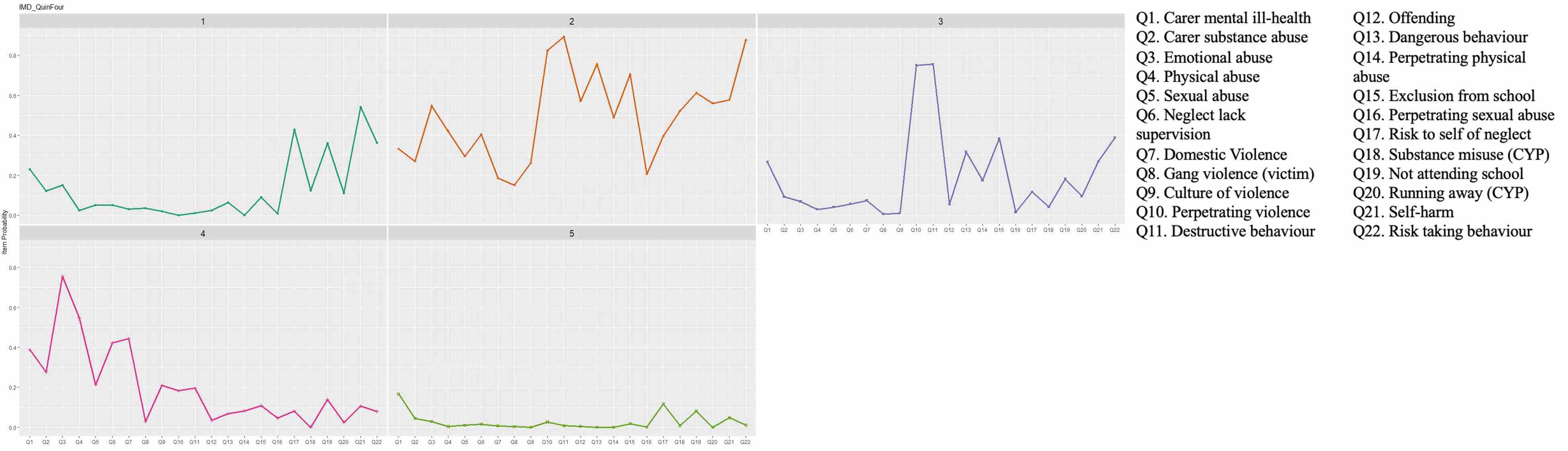


S34 Profile plots IMD Quintile 5^th^


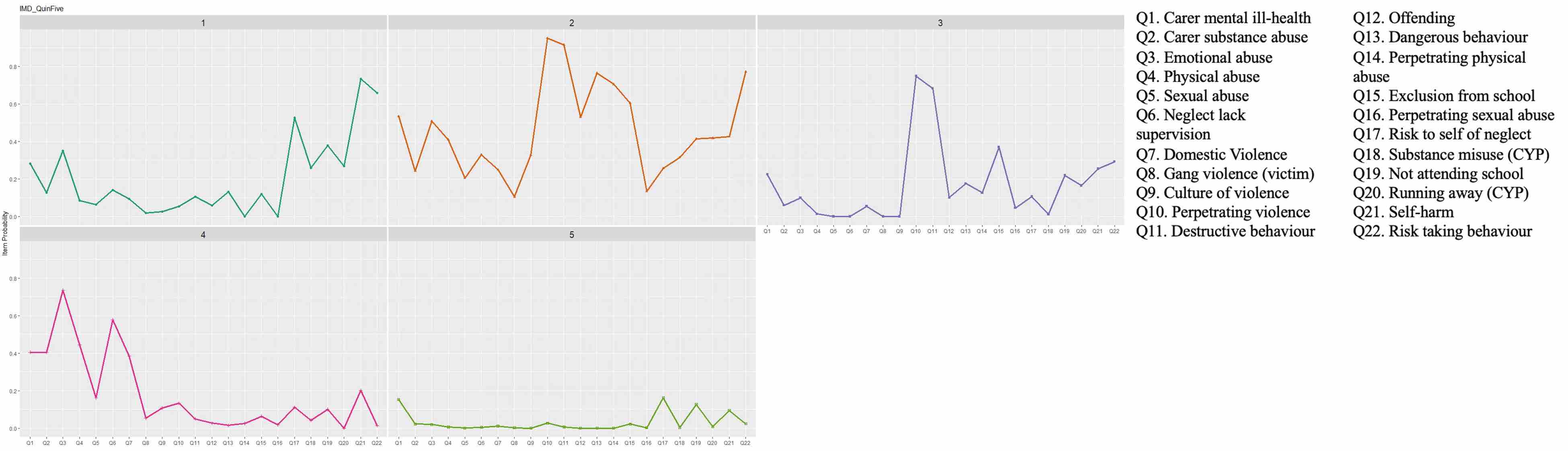


S35 Profile plots No Child protection involvement


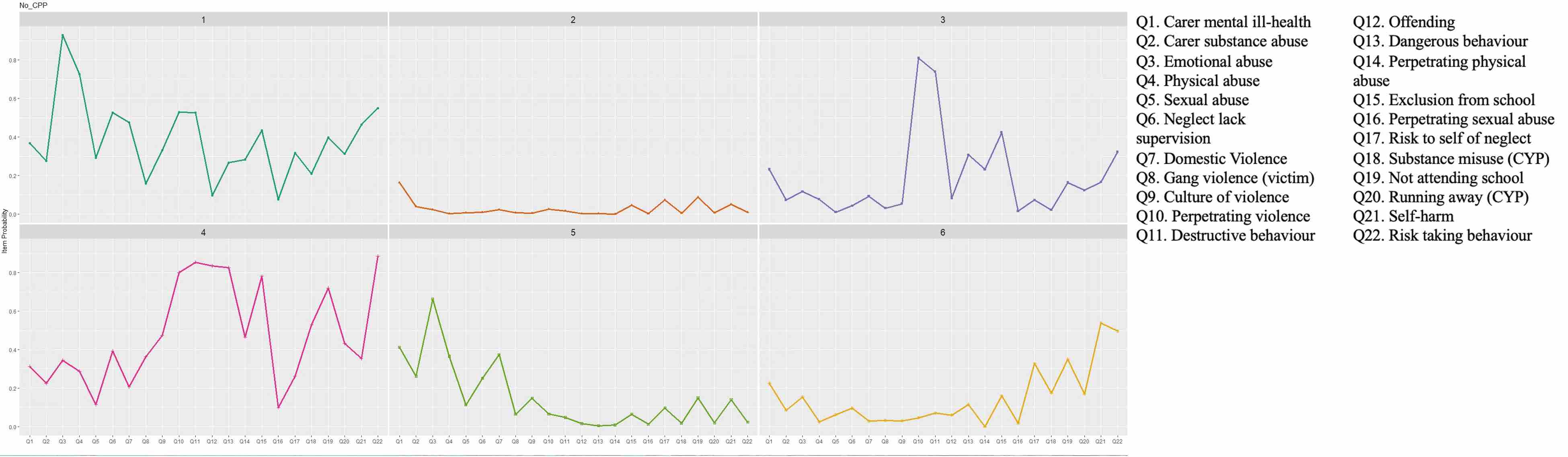


S36 Profile plots Past or Present Child Protection Involvement


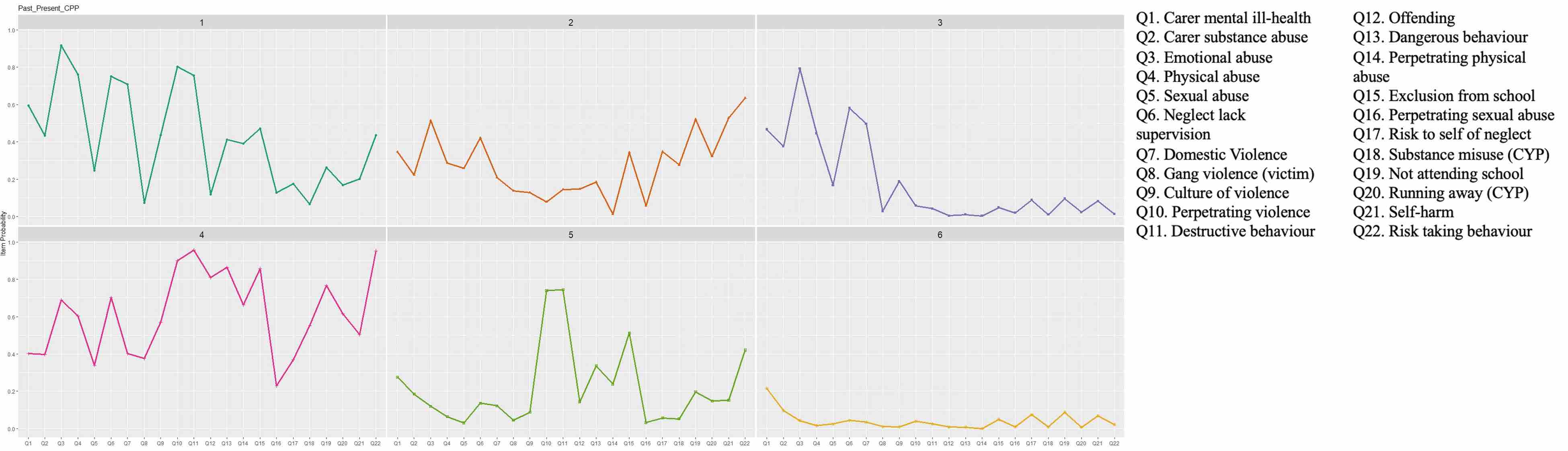


S37 Goodness of fit subset (n=16299)

|  | loglik | Gsq | df | AIC | CAIC | BIC |
| --- | --- | --- | --- | --- | --- | --- |
| 2 | -116103.87 | 51782.02 | 16253 | 232297.74 | 232689.18 | 232644.18 |
| 3 | -112561.25 | 44696.78 | 16230 | 225258.49 | 225850.01 | 225782.01 |
| 4 | -110021.06 | 39616.41 | 16207 | 220224.12 | 221015.72 | 220924.72 |
| 5 | -108982.08 | 37538.45 | 16184 | 218192.16 | 219183.83 | 219069.83 |
| 6 | -108333.83 | 36241.95 | 16161 | 216941.67 | 218133.41 | 217996.41 |
| 7 | -107852.34 | 35278.97 | 16138 | 216024.68 | 217416.5 | 217256.5 |

k = Number of classes; loglik = maximum log-likelihood; Gsq = likelihood-ratio/deviance statistic; df = residual degrees of freedom; AIC = Akaike information criterion; CAIC = Bozdogan's criterion; BIC = Bayesian information criterion

S38 Profile plots subset (n=16,299)


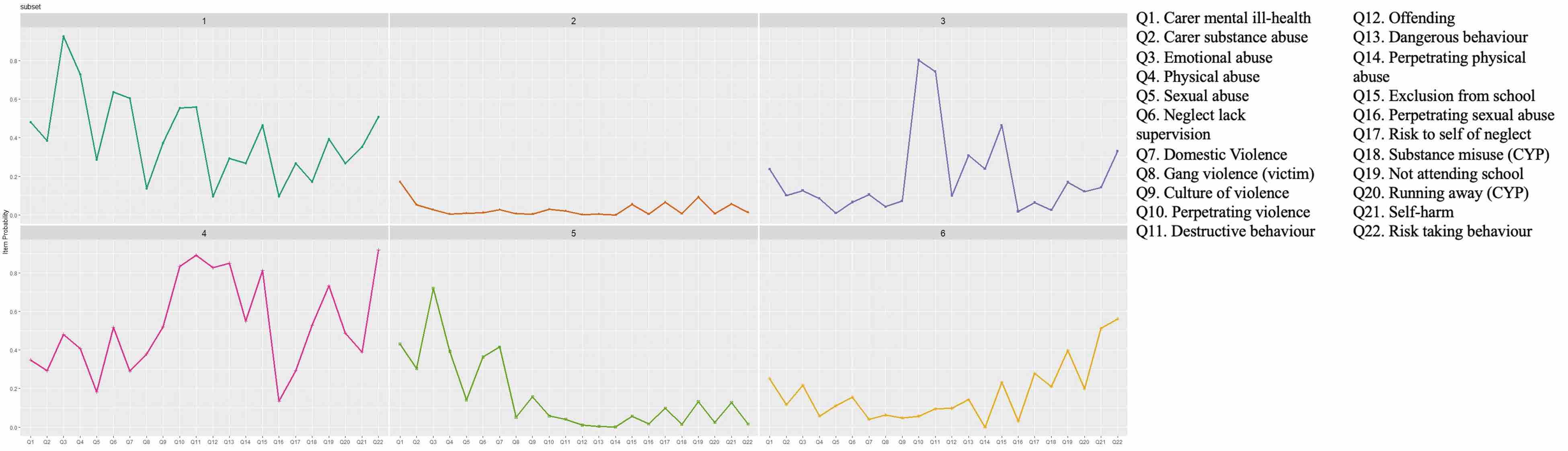

Supplement: Supplementary file 1 — Supporting Information S1 [file JCV2-4-e12246-s001.docx]
